# Bacoside A Induces Tumor Cell Death in Human Glioblastoma Cell Lines through Catastrophic Macropinocytosis

**DOI:** 10.3389/fnmol.2017.00171

**Published:** 2017-06-15

**Authors:** Sebastian John, K. C. Sivakumar, Rashmi Mishra

**Affiliations:** ^1^Disease Biology Program, Department of Neurobiology and Genetics, Rajiv Gandhi Centre for BiotechnologyThiruvananthapuram, India; ^2^Distributed Information Sub-Centre, Rajiv Gandhi Centre for BiotechnologyThiruvananthapuram, India

**Keywords:** biomechanics of cancer, CaMK2A, glioblastoma, *Bacopa monnieri*, Bacoside A, drug-repurposing, macropinocytosis, non-apoptotic cell death

## Abstract

Glioblastoma multiforme (GBM) is a highly aggressive type of brain tumor with an extremely poor prognosis. Recent evidences have shown that the “biomechanical imbalances” induced in GBM patient-derived glioblastoma cells (GC) and *in vivo* via the administration of synthetic small molecules, may effectively inhibit disease progression and prolong survival of GBM animal models. This novel concept associated with *de novo* anti-GBM drug development has however suffered obstacles in adequate clinical utility due to the appearance of unrelated toxicity in the prolonged therapeutic windows. Here, we took a “drug repurposing approach” to trigger similar physico-chemical disturbances in the GBM tumor cells, wherein, the candidate therapeutic agent has been previously well established for its neuro-protective roles, safety, efficacy, prolonged tolerance and excellent brain bioavailability in human subjects and mouse models. In this study, we show that the extracts of an Indian traditional medicinal plant *Bacopa monnieri* (BM) and its bioactive component Bacoside A can generate dosage associated tumor specific disturbances in the hydrostatic pressure balance of the cell *via* a mechanism involving excessive phosphorylation of calcium/calmodulin-dependent protein kinase IIA (CaMKIIA/CaMK2A) enzyme that is further involved in the release of calcium from the smooth endoplasmic reticular networks. High intracellular calcium stimulated massive macropinocytotic extracellular fluid intake causing cell hypertrophy in the initial stages, excessive macropinosome enlargement and fluid accumulation associated organellar congestion, cell swelling, cell rounding and membrane rupture of glioblastoma cells; with all these events culminating into a non-apoptotic, physical non-homeostasis associated glioblastoma tumor cell death. These results identify glioblastoma tumor cells to be a specific target of the tested herbal medicine and therefore can be exploited as a safe anti-GBM therapeutic.

## Introduction

Cancer cells demonstrate high tunability to various microenvironmental physical forces such as the hydrostatic pressure from the interstitial fluid flow, shear forces in the blood vessels, rigidity stresses from the extracellular matrices, compressive stresses from the extracellular proton concentrations and the osmotic stresses from ionic changes, all of which stimulates tremendous genetic/epigenetic survival maneuverings, tumor heterogeneity and malignancy (Suresh, 2007; Brocker et al., 2012; Li and Hanahan, 2013; Asghar et al., 2015; John et al., 2017; Lee et al., 2017).

Glioblastoma multiforme (GBM) is the most aggressive form of brain tumors with a mean survival time ranging from a few months to 3 years and temozolomide, an only FDA approved drug for GBM treatment, is facing large setbacks due to the ever growing tumor heterogeneity, quiescent but re-activable pool of tumor initiating cells and chemoresistance (Kitambi et al., 2014). Hence, there is a strong need to explore newer avenues for anti-GBM drug therapeutics that can hijack tumor cells’ higher organizing principles such as the biomechanical homeostasis.

Recent studies in this direction report that a clever design of synthetic small molecules such as Vacquinol-1 can hijack tumor cell “hydrostatic balance” by induction of an excessive cell drinking/“methuosis”/macropinocytosis leading to tremendous fluid accumulation and rise in membrane tension causing loss of membrane integrity/cell lysis associated death of heterogeneous glioblastoma tumor niches (including tumor initiating cells) in both *in vitro* and *in vivo* tumor models (Overmeyer et al., 2011; Kitambi et al., 2014). This “macropinocytosis induced” new mechanism of rendering GBM cells vulnerable to cell death is highly interesting but the studies did report evidences of non-specific or unrelated toxicity upon prolonged administration of the synthetic molecule.

Macropinocytosis or excessive cell drinking is enabled by actin-driven large membrane buckling forces and is shown to be promoted by intracellular calcium *via* the Ras/Rac1 pathway (Aspenström, 2004; Falcone et al., 2006; Overmeyer et al., 2008; Kabayama et al., 2009; Egami et al., 2014; Ha et al., 2016). Hence, we designed a protocol by which we aimed to successfully induce higher calcium levels, specifically in tumor cells, by an alternative natural product based strategy which was already demonstrated to be safe even on prolonged dosing to human subjects.

Tumor cells are known to express a crucial kinase, calcium/calmodulin-dependent protein kinase II (CaMKII/CaMK2), and its phosphorylation essentially triggers high calcium release from the ryanidone channels of the ER for various tumor associated metabolic and adaptive processes (Ozawa, 2010; Wang et al., 2015). CaMK2 modulations (mainly “inhibition”) are therefore being exhaustively researched for anti-tumor therapeutics in breast, prostate, osteosarcoma, liver and CML cancers though its targeting in brain cancers is not yet robustly studied (Li and Hanahan, 2013; Pellicena and Schulman, 2014; Wang et al., 2015; Chi et al., 2016). However, since this enzyme is a crucial component of synaptic plasticity, learning and memory processes, muscle and cardiac functioning; its “inhibition”/suppression can generate severe cognitive problems on one side and malfunctioning of cardio-muscular system on the other (Lisman et al., 2012; Wang et al., 2015; Chi et al., 2016). Therefore in this study, instead of inhibiting, we rather tried to “enhance” tumor specific phosphorylation of CaMK2A in Glioblastoma cells (GC) *via* the administration of *Bacopa monnieri* (BM) extract components as well as its major bioactive component Bacoside A as these are established phosphorylation activators of CaMK2A (Prisila Dulcy et al., 2012; Le et al., 2013) and can potentially promote the release of high intracellular calcium that may result in excessive cell drinking and hydrostatic plasma membrane stress mediated tumor cell lysis, akin to Vacquinol-1 (Ozawa, 2010; Kitambi et al., 2014). It is to be noted that inhibition of phosphorylation of CaMK2A was recommended to be beneficial in breast cancer progression (Chi et al., 2016). However, bacopa and bacoside A which enhance CaMK2A phosphorylation, are also demonstrated to exert excellent cytotoxic effects on breast cancer cells, in hepatocarcinogenesis etc. (Janani et al., 2010; Prakash et al., 2011; Nandagaon and Kulkarni, 2013; Yadav and Reddy, 2013; Jose et al., 2014; Patil et al., 2014; Mallick et al., 2015). Hence, these reports overall suggest that disturbances in the homeostatic levels of phospho CaMK2A (either by inhibition or *via* enhancement) may turn tumor cell vulnerable to death.

## Materials and Methods

### Chemicals and Cell Lines

All chemicals were mainly purchased from Sigma Aldrich, St. Louis, MO, USA, unless otherwise specified. Bacoside A [3-((alpha)-L-arabinopyranosyl)-O-(beta)-D-glucopyrano-side-10,20-dihydroxy-16-keto-dammar-24-ene] was from Natural Remedies Pvt. Ltd, Bangalore, India^1^. BM (brand name: Brahmi) was from The Himalaya Drug Company, Bangalore, India^2^. Calmodulin Binding Domain was from Calbiochem (CAMBD, cat no. CAS 115044-69-4), Dextromethorphan (DXM, cat no. D2531) and 1,2-Bis(2-Amino-5-methylphenoxy)ethane-N,N,N′,N′-tetraacetic acid tetrakis (acetoxymethyl ester) [BAPTA-AM, cat no. 16609] were from Sigma; X-Rhod-1-AM, Fura-2AM, Dextran-TMR and Rhodamine Phalloidin were from Invitrogen. All cell culture reagents were from Invitrogen. Antibodies used were as follows: LAMP1, Rab7, CAV1, phospho CAV1 Y14, NR2B, phospho NR2B, CREB1, phospho CREB1, K-Ras, LC3II, all from Cell Signaling Technology, Danvers, MA, USA. CaMK2A (ab52476) and phospho-CaMK2A T286 (ab171095) were purchased from Abcam, Cambridge, MA, USA. *In situ* cell death detection kit was from Roche Applied Science. GBM patient tissue array was from US Biomax Inc. (cat no.GL805a). All human glioblastoma cell lines, namely, LN229, U87MG and U251 were purchased from ATCC, Manassas, VA, USA. Human Subventricular radial glial progenitor (SVG) cell line was gifted by Prof. Pankaj Seth from the National Brain Research Centre, Manesar, India and generated as described in Messam et al. (2003). Human normal cell line HaCaT (epidermal keratinocytes) was purchased from ATCC, USA. Please note that most of the initial standardizations were performed on LN229 and to further test the robustness and reproducibility of the observation, U87MG and U251 glioblastoma lines were used.

### Cell Culture, *Bacopa monnieri*, Bacoside A and Inhibitor Treatments

All cells were seeded at a density of 7 × 10^4^ cells in eight well chamber slides with DMEM (high glucose, 25 mM) + 10% FBS + 1X antibiotic for 16 h in 5% CO_2_ at 37°C. LN229 glioblastoma cells, human normal cell lines, SVG and HaCaT, were given respective treatments with BM (dissolved overnight in medium and filtered to separate the undissolved fractions, dosages 0–150 μg/ml were administered); Bacoside A was dissolved in ethanol and used in concentrations ranging from 0–8 μg/ml on LN229 glioblastoma cell line. The standardized dose was further tested in U87MG and U251 glioblastoma cell lines. The cell morphological changes in response to treatments were monitored live at various time intervals using 10× and 20× magnification objectives fitted on Olympus 1X71 microscope. For long duration experiments, the compounds were replenished every 12 h. The inhibitor treatments were performed on LN229 and U87 MG glioblastoma cell lines at the following working concentrations: CAMBD (200 nM), BAPTA (20 μM), DXM (10 μM). Briefly, the inhibitors were loaded on the cells in full growth medium for at least 2 h and then either the cultures were replaced with the fresh inhibitors alone or with Bacoside A at a concentration of 8 μg/ml.

### Acridine Orange-EtBr Live/Dead Assay

The assay was performed on LN229 and U87MG glioblastoma cell lines according to the protocols described in Mironova et al. (2007).

### Tunel Assay

The assay was performed on LN229 and U87MG glioblastoma cell lines using Roche “*In situ* cell death detection kit” according to the manufacturer’s instructions.

### Cell Proliferation/Viability Assay

Live/Dead Viability kit was purchased from Thermo Fisher Scientific/Molecular Probes, Eugene, OR, USA. This Calcein-AM/EtBr based cytotoxicity and viability assay was performed on LN229, SVG and HaCaT cell lines according to the manufacturer’s instructions.

### Calcium Imaging

(i) Fura 2AM calcium imaging on LN229 glioblastoma cell line was performed as described in Li et al. (2011). Briefly, cells were seeded in 96 well platforms. At the end of the experiment, post 12–24 h treatment, cells were washed in warm IX HBSS and Fura2AM dye was used at a concentration of 4 μM with 0.04% Pluronic-F127. Cells were then incubated in dark for 30 min at room temperature. The cells were further washed and kept in dark for additional 15 min. After a quick wash, the ratiometric reading of signal was performed in TECAN M200 multiplate reader with the following settings: Excitation: 340 nm and 380 nm, Emission: 510 nm. The continuous fluorescence kinetics was performed for 30 min and results were plotted for an “average reading” over each kinetics cycle done in triplicate. (ii) X-Rhod1-AM based calcium imaging probe was used to detect free calcium in LN229 and U87MG glioblastoma cell lines according to the manufacturer’s instructions (Ex/Em = 508/602 nm). 100 μl of 5 μM X-Rhod-1 AM was applied in HBSS at 37°C, 5% CO_2_ for 1 h. Cells were washed two times with HBSS and imaged using TRITC channel for 1 min.

### Real Time PCR

mRNA was extracted from LN229 glioblastoma cultured cells with Qiagen RNAeasy kit and reverse transcription into cDNA was performed with AB biosystem cDNA synthesis kit. RT PCR was performed using SYBR green method as described in the user’s protocol of AB Biosystems real time PCR reagents and AB Biosystem Real Time PCR user’s manual. Comparative *C*_T_ method was used for analyzing Real Time PCR Data. Human gene Primer sequences were chosen from qPrimerDepot database^3^.

CaMK2A:
Right primer sequence: TGATCTTGGCAGCATACTCCT
Left primer sequence: CGCTTCACGGAAGAGTACCA
SOD1:
Right primer sequence: CCACACCTTCACTGGTCCAT
Left primer sequence: CTAGCGAGTTATGGCGACG
RIPK1:
Right primer sequence: TCACAACTGCATTTTCGTTTG
Left primer sequence: GGCATTGAAGAAAAATTTAGGC
RIPK3:
Right primer sequence: GTTGTCTCCAACTTGCACCC
Left primer sequence: ACAAGGCATGAACTGGTCCT
UBA52:
Right primer sequence: CTCAATGGTGTCACTGGGC
Left primer sequence: TTCTTTTTCTTCAGCGAGGC

### Western Blotting

Western blots were performed on LN229 glioblastoma cultured cells as described in Sadowski et al. (2013).

### Lipid Raft Isolation

Detergent resistant membranes were prepared as follows: LN229 glioblastoma cells were cultured in T175 flask, medium was discarded and cells were washed in cold PBS. The cells were then incubated for 45 min in 4 ml of cold PBS buffer containing 1% Triton X-100, 0.01% PMSF and protease inhibitors (using Complete Mini Protease inhibitor tablet, Roche Diagnostics, Mannheim, Germany) at 4°C. The cells were collected and suspension was homogenized (with 20 manual strokes using glass pestle and motor with grinded bottom) in cold room. Cell homogenate (3 ml) was mixed with equal volume of 90% sucrose solution and was overlaid with 3 ml 35% sucrose and 3 ml 5% sucrose in 12-ml ultracentrifuge tube. The samples were centrifuged at 41,000 rpm in a Beckman L8-80 M ultracentrifuge using SW41Ti rotor for 16 h at 4°C. After centrifugation, a thin white band was visible at 5%–35% sucrose density interface containing the detergent-resistant light membrane fraction (fraction 3, 4, 5 from top; each fraction was of 1 ml). This fraction was retrieved and used for detection of raft associated caveolin-1 and non-raft associated K-ras proteins. The lower fractions 8–12 (non-detergent resistant) were used for quantitation of non-raft proteins *via* the dot blot assay. Protein quantitation for equal loading was performed using BCA protein assay kit (Sigma, St. Louis, MO, USA).

### Immunohistochemistry on GBM Patient Tissue Array

The immunohistochemistry was performed according to John et al. (2017). CaMK2A antibody (ab52476) predominantly detects the non-phosphorylated protein pool of CaMK2A (non-phospho peptide around the phosphorylation site T286 was used to raise antiserum) and pCaMK2A antibody (ab171095) specifically recognizes the phosphorylated CaMK2A at T286/287 residue.

In order to further ensure the detection of phospho *vs.* non-phospho CaMK2A signal in IHC and in immunocytochemistry based experiments, first phospho CaMK2A IHC/ICC was performed and the signal was fluorescently developed. This masked the major phosphorylated epitopes. Non-phospho CaMK2A antibody was then used to detect the rest of the available CaMK2A pool. Please note that pCaMK2A antibody was rabbit monoclonal and CaMK2A antibody was mouse monoclonal.

### Immunocytochemistry and F-actin Staining

It was performed on LN229 and U87MG glioblastoma cell lines according to John et al. (2017).

### Dextran TMR-Fluid Phase Uptake

8 × 10^4^ cells were plated in 8-well chamber slide containing DMEM, 10% FBS and 1X antibiotic. LN229 and U87MG glioblastoma cell lines were BM or Bacoside A treated as discussed earlier. Post 4 h of treatment, when phase lucent macropinosomes were detected under phase contrast microscope, the cells were incubated with 2 mg/ml Dextran TMR in the treatment medium. Post incubation, the cells were washed with PBS with calcium and magnesium and were fixed with 1.5% PFA. At least 200 cells from five random fields, in three independent experiments, were imaged using Nikon A1R confocal microscope and the intensity was quantitated using Fiji Image analysis software.

### pH Gradient Assay for Compound Functionality

pH gradients were generated on LN229 glioblastoma cell line as described in John et al. (2017) and Bacoside A treatment was performed. Live cell monitoring at various time points was done to probe the efficiency of Bacoside A vacuolization dependent cytotoxicity in various pH microenvironments.

### Single Cell Analysis

Single cell analysis for protein expression quantitation was performed according to John et al. (2017). Briefly, protein profile of the cells were derived by using free hand tool ROI followed by “Measure” application in Fiji software. At least 200 cells, from five random fields were analyzed. The experiment was repeated thrice and average of each experiment was plotted in a graph with display of error bars (SD) and significance of comparisons (*p*-values).

### Glide Score Calculations

Glide score calculations was performed using Glide feature from Qualified Non-Commercial User licence version of Schrodinger Suite 2016-I provided to KCS. GlideScore is a measure of approximate ligand binding free energy; and a GlideScore of −7.0 and below suggests high ligand binding affinity. Molecular Docking and simulations were performed with the same software. Panel B in Figure 6 was generated using PyMol v1.1.

**Figure 1:**
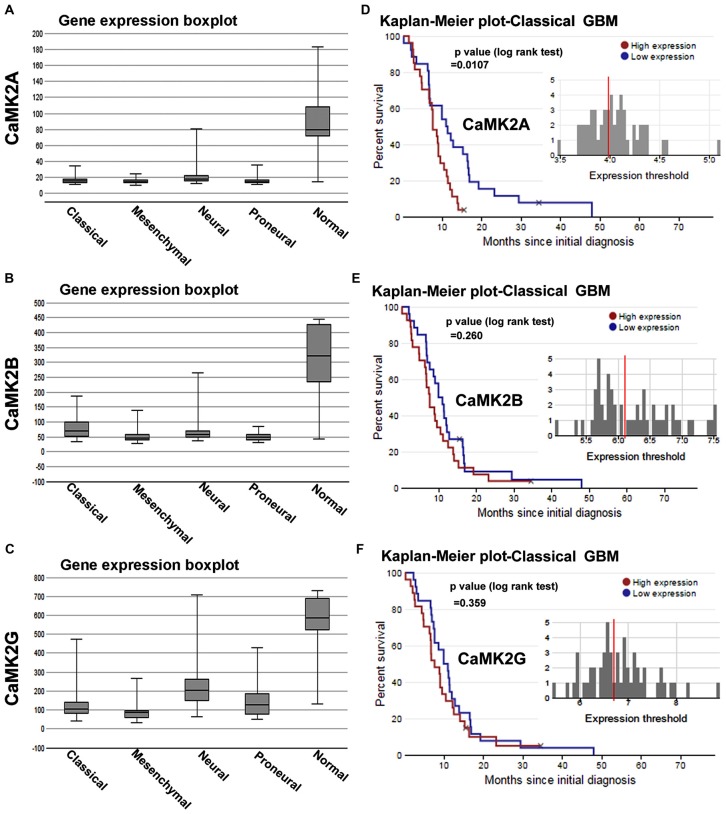
Calcium/calmodulin-dependent protein kinase II (CaMKII) gene expression and survival curves are associated with lower survival rates in glioblastoma multiforme (GBM) patient sub-types. **(A–C)** shows The Cancer Genome Atlas (TCGA) data based comparative gene expression profiles of CaMK2A, 2B and 2G isoforms of CaMK2 enzyme in normal and glioblastoma patients, sub-classified into classical, mesenchymal, neural and proneural GBMs. Data revealed that all isoforms of CaMK2 are expressed at a lower levels in GBM than in normal controls. CaMK2G gene expression was highest in all subtypes in comparison to other isoforms. **(D–F)** respectively shows CaMK2A, 2B and 2G associated Median Survival Curve analysis using glioblastoma patient data in TCGA data set under classical (most predominant) GBM subtype. Observations revealed that CaMK2A expression alone was significantly associated with poor prognosis [median survival, 15 months in CaMK2A high group (red) vs. 48 months in CaMK2A low group (blue)]. See also Supplementary Figures S1, S2 and S3 for more details on expression and survival curves in other GBM subtypes. TCGA data was extracted from URL: www.betastasis.com.

**Figure 2:**
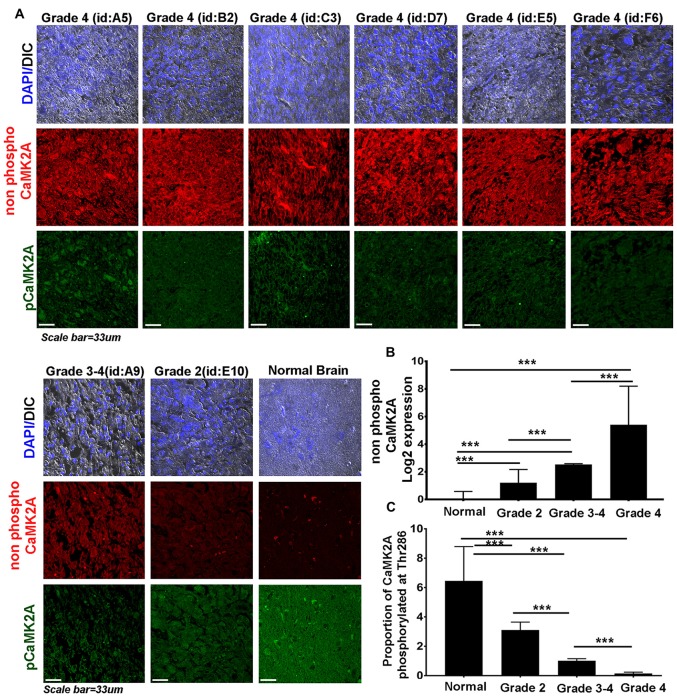
CaMK2A phosphorylation at T286 is reduced in more advanced stages of GBM tumors. **(A)** Immunohistochemistry (IHC) based comparative and quantitative survey of the non-phosphorylated CaMK2A and its phosphorylation at T286 was performed using glioblastoma patient tissue array with 63 samples from WHO Grade IV (including four pediatric samples, Supplementary Figure S4), three samples from Grade III, four samples from Grade II and six normal controls. The identities of representative samples from each tumor grade as mentioned in the tissue array are specified. **(B)** Data shows that significantly higher levels of non-phosphorylated CaMK2A protein levels were associated with increase in GBM grade but **(C)** reverse trend was noticed for the proportion of CaMK2A phosphorylation at T286, which was determined by normalizing the levels of phospho-T286 to non phosphorylated CaMK2A. This suggests that phosphorylation of CaMK2A was tightly regulated in progressive GBM tumor grades and was crucially kept at lower levels. It is to be noted that normal brain samples expressed very high levels of phospho CaMK2A T286. ***p < 0.001. Error bar = SD.

**Figure 3:**
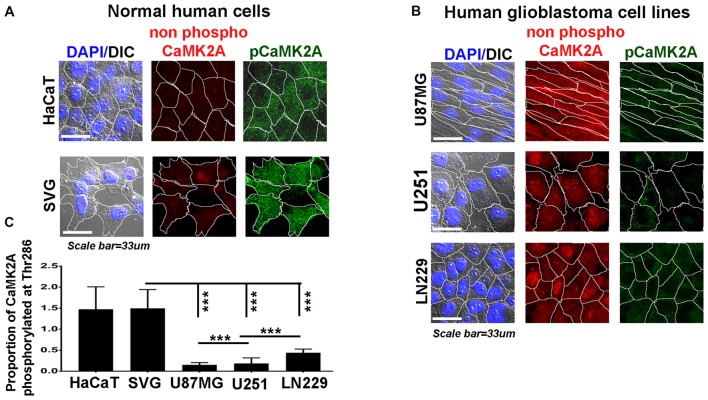
Levels of CaMK2A phosphorylation at T286 is lower than non-phospho CaMK2A in aggressive GBM tumor cell lines *vs.* normal cells. Single cell quantitative image analysis procedure (see John et al., 2017) was employed to measure non-phospho CaMK2A and pCaMK2A T286 protein levels in **(A)** normal human cells: SVG-subventricular zone derived glial cells; HaCaT-epidermal keratinocytes and **(B)** various GBM patient derived tumor cell lines: U87MG, U251 and LN229. **(C)** The proportion of phosphorylated CaMK2A determined by normalizing average signal of phospho CaMK2A T286 (obtained from over 200 cells in five random fields, per three independent experiments) to average signal from non-phospho CaMK2A showed higher levels in normal cells and significantly lower levels in GBM cell lines. The results corroborate well with the observation on GBM patient tissue array. The analysis is representative of three independent experiments. ***p < 0.001. Error bar = SD.

**Figure 4:**
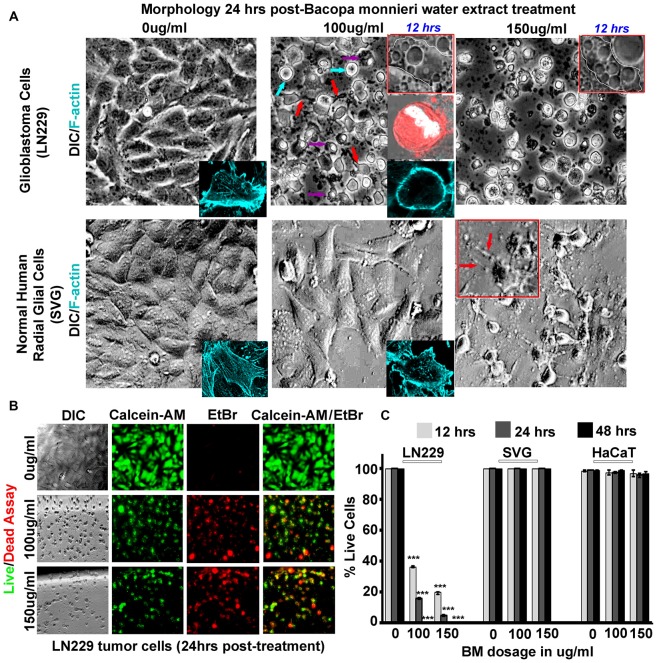
*Bacopa monnieri* (BM) a known inducer of CaMK2A phosphorylation generates glioblastoma cytotoxicity. **(A)** BM extract, an inducer of CaMK2A phosphorylation, was administered to LN229 glioblastoma tumor cell line cultures in various doses and monitored at various time points. Human normal radial glial cells (SVG) were used as normal controls. Top insets in middle and last panels of LN229 shows that BM treatments (100 μg/ml and 150 μg/ml), within 12 h, induced phased lucent enlarged vacuoles in cells, characteristic of macropinosomes, *also see* Supplementary Figure S5A for confirmation of macropinocytotic vacuoles. Second inset in the middle panel of LN229 shows Dextran TMR (red, which is uptaken by macropinosomes and released into the cytoplasm) filled, rounded and swelled tumor cell with squashed nucleus (white, due to raised hydrostatic pressure of intracellular fluid) in 24 h post treatment, please see Supplementary Figure S5B for more detailed images. Last inset shows loss of cell actin cables (rhodamine phallodin staining shown in cyan) due to cell swelling. The comparative analysis of main panels on the basis of morphology and F-actin staining suggests no cytotoxicity to normal cells, however, LN229 cells showed cell rounding (cyan arrows), cell swelling, nuclear compression (magenta arrows) and cell necrosis (red arrows), post 24 h of treatment. A dose of 150 μg/ml showed SVG differentiated phenotype, hence the therapeutic dose was determined to be 100 μg/ml. **(B,C)** Calcein-AM (green, live cell marker probe)/EtBr (red, damaged and dead cell marker dye) based cytotoxicity and viability analysis over various time points (12, 24 and 48 h) post BM treatment at 0, 100 and 150 μg/ml showed specific cytotoxic effects on LN229 glioblastoma cell lines *vs.* the normal cells (SVG and HaCaT). ***p < 0.001. Error bar = SD The analysis is representative of three independent experiments.

**Figure 5:**
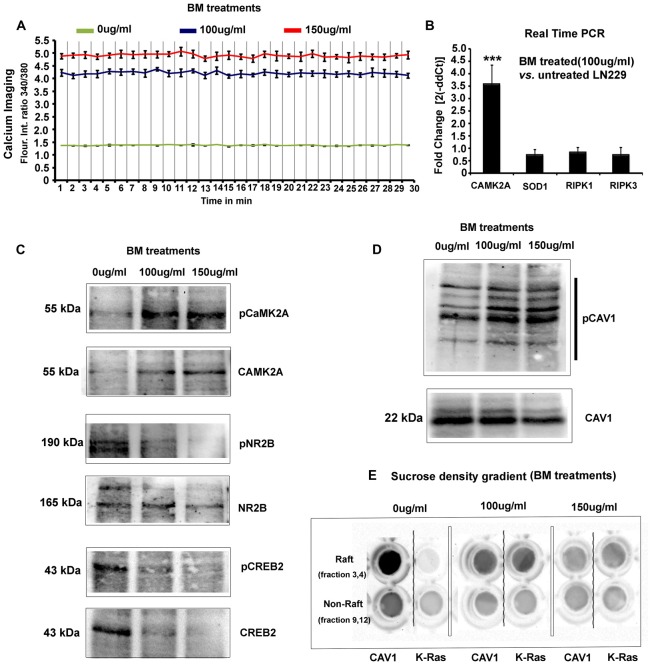
BM induces imbalances in glioblastoma tumor cells physico-chemical homeostasis involving pCaMK2A T286-Ca^2+^ axis. **(A)** As phosphorylated CaMK2A is known to trigger intracellular calcium release, free calcium flux, upon BM treatment were measured by Fura2-AM dye based ratiometric imaging. Results showed a 3–3.5 fold higher levels of free calcium in treated *vs.* untreated LN229 Glioblastoma cells (GC). **(B)** Real time PCR analysis to measure the fold change in the transcripts of CaMK2A (to measure the effects of BM on CaMK2A at a gene expression), SOD1 (to measure the tumor cell survival response to high calcium induced oxidative stress) and RIPK1/RIPK3 (to measure possible induction of necroptosis) was performed after 24 h of BM treatment at a dose of 100 μg/ml. Significant increase in CaMK2A transcript *vs.* untreated LN229 glioblastoma tumor cells showed that BM acts by not only phosphorylating but also by increasing the pool of CaMK2A enzyme that can be phosphorylated. **(C)** Western blot analysis of the levels of pCaMK2A T286 *vs*. non-phospho CaMK2A upon BM treatment corroborates with the real time PCR results and shows that the levels of unphosphorylated CaMK2A is enhanced upon BM treatment and so does the pool of phosphorylated CaMK2A in treated *vs.* untreated conditions. This phosphorylation was unlikely due to the activation of NMDA-pCaMK2A pathway as major downstream signaling components, pNR2B and pCREB showed rather reduced levels in BM treated *vs.* untreated tumor cells. **(D)** Total cellular levels of CAV1 protein, also a marker of caveolae, showed a decrease at 150 μg/ml but its Y14 phosphorylated form, which is essentially the CAV1 pool destabilized from the surface due to disassembly of caveolae showed a significant increase, suggesting that BM treatment generated higher membrane tension leading to loss of surface tension buffering caveolar reserves. **(E)** Sucrose density gradient combined with dot blot based determination of the levels of surface raft/caveolar caveolin *vs.* non raft/non-caveolar caveolin (fractions with non-raft marker protein K-RAS), showed that with BM treatment, less CAV1 protein was identified in raft fractions, suggesting a loss of caveolae/lipid rafts the from surface. ***p < 0.001. Error bar = SD The analysis is representative of three independent experiments.

**Figure 6:**
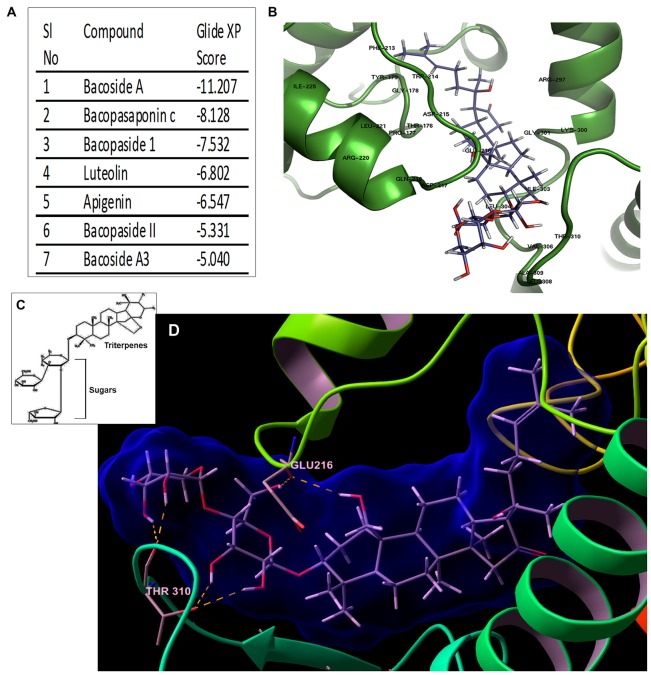
Bacoside A—the bioactive component of BM binds to CaMK2A *in silico*. **(A)** Binding affinity (glide score) of different components of the extract of BM to CaMK2A activation domain (200–310 amino acid residues) showed highest glide ratio for Bacoside A which is also the established active component of BM. **(B)** shows a docked model of CaMK2A-Bacoside A. See Supplementary Figure S6 for docking of other components of BM to CaMK2A. **(C)** shows the amphipathic molecular structure of Bacoside A constituting hydrophilic glycans and lipophilic terpenoid moieties **(D)** shows Bacoside A sugar moieties makes hydrogen bonds interactions with CaMK2A protein residues namely Glu216 in the Ser/Thr catalytic domain (13–271 amino acids) and Thr310 in the association domain (293–310 amino acids). Also see Supplementary Figure S7 for more resolved image of the glycan-amino acid interaction in Bacoside A-CaMK2A docked model. Arabinose is observed to interact with Thr310 and Glu216, whereas glucose interacts with Thr310.

### Microscopy

Confocal and DIC microscopy was performed according to John et al. (2017). Please see supplementary datasheet 1 of the mentioned reference for more details.

### Clinical Data Analysis

Detailed patient data were obtained from the The Cancer Genome Atlas (TCGA) REpository for Molecular BRAin Neoplasia DaTa (REMBRANDT) databases publicly available at the Project Betastasis website^4^. The graphical representation of gene expression data for CaMK2 isoforms in different glioma subtypes were downloaded from the REMBRANDT. The transcriptome analysis and Gene survival association (Affymetrix HT HG U133A) for Glioblastoma subtypes were downloaded from TCGA portal in betastasis^4^. The patient survival curves and corresponding Log rank test *p*-values were obtained by setting “median range” of survival in 453 patients.

### Statistics

All above experiments were performed in triplicates. Each independent experiment had three technical replicates. Error bars are indicative of average standard deviations (SD) obtained across three independent experimental sets. The *p* values were obtained through Bonferroni’s *t*-test between controls and respective treatment conditions and were represented as one star, two stars or three stars to denote *p* ≤ 0.05, 0.01, 0.001 respectively. In individual situations, other comparisons of significance were also indicated. Image analysis was done on over 200 cells in each condition using Fiji image processing software. Calibration bar for LUT converted images were shown in the respective figures. The importance of LUT converted image is explained in John and Mishra (2016).

## Results

### CaMK2A Expression Predicts Poor Prognosis in Glioblastoma Cancer Patients

We first analyzed the mRNA expression levels of CaMK2 isoforms in glioma patients from a publically available database (data obtained from TCGA, *see “Materials and Methods”* Section and www.betastasis.com). Extracted data showed that CaMK2A, 2B and 2G were expressed at “lower” and 2D at comparable levels in gliomas (sub-classified into GBM, oligodendroglioma and astrocytoma) in comparison to the normal subjects (Supplementary Figure S1). In GBM-glioma subtype the order of expression from highest to lowest was as follows: CaMK2G > CaMK2B ≥ CaMK2A > CaMK2D (Supplementary Figure S1).

Within the GBM subtypes, i.e., Classical, Proneural, Mesenchymal and Neural, all CaMK2 isoforms showed overall expression to be “comparatively lower than normal subjects”. The order of expression of CaMK2 isoforms in classical GBM, the most abundant GBM, was as follows: CaMK2G > CaMK2B > CaMK2A (highest to lowest; Figure 1).

However, only CaMK2A associated patient survival curves with classical GBM was significant, and showed that high expression of this isoform was associated with poor prognosis (*p* = 0.0107; Supplementary Figures S2, S3). CaMK2G high expression was found to be significantly associated with poor prognosis of neural subtype (*p* = 0.0262), which infrequently occurs in old age (Supplementary Figure S3).

### High Grade Glioblastomas Were Associated with Low Phospho-CaMK2A Levels

Even though high CaMK2A transcript level was found to be associated with poor survival of GBM patients, the co-relation of the phosphorylated state of CaMK2A protein with patient survival was not evident.

Therefore, we examined the protein levels (*via* immunohistochemistry) of non-phospho and T286 phosphorylated CaMK2A in glioblastoma patient tissues array which had samples from different grades (astrocytoma-grade 2, 3–4 and multiforme Grade 4) and normal brain tissues. Results showed that GBM (grade IV) had higher levels of non-phosphorylated CaMK2A *vs.* the lower grades, suggesting that a regulated “phosphorylation inhibition of CaMK2A” was essential for glioblastoma progression to aggressive and malignant form (Figure 2). The results were found to be consistent with the observations made on pediatric GBMs that are essentially attributed with different mutational landscape than adult GBMs (Supplementary Figure S4). A panel of normal and glioblastoma tumor cell lines were next examined for the expression of non-phospho and phospho CaMK2A-T286 and the immunofluorescent images were further quantitated using ImageJ software (Figure 3). The results were in high concordance with patient data as GBM tumor cell lines showed higher non-phospho CaMK2A levels *vs.* its phosphorylated form. It is to be noted that normal cell lines showed much higher levels of pCaMK2A-T286 in comparison to GBM cells, which was also observed by tissue histochemistry. Hence, a chemical that would over-ride this phosphorylation suppression could make GBM tumor cells vulnerable to cell death by excessive cellular calcium release, essentially due to the documented action of pCaMK2A on calcium releasing ryanidone channels.

In this context, we proceeded to examine the probable anti-GBM effects and mechanisms of action of BM extract and its bioactive component Bacoside A, as these are demonstrated to be the potential regulators of CaMK2A phosphorylation (Prisila Dulcy et al., 2012; Le et al., 2013; Figures 2, 3).

### *Bacopa monnieri* (BM, a Regulator of CaMK2A Phosphorylation) Whole Plant Extract Causes Rapid and Specific Loss of Viability of GBM Tumor Cells

To gain “first mechanistic insights” into the prospects of Bacopa (BM) induced GBM tumor cell death, we initially focused on only one human GBM tumor cell line and subsequently expanded the observation to other GBM tumor cells. The well-established GBM cell line, LN229 was screened for cytotoxicity with dosages in the range of 0–150 μg/ml and the results were filtered against human sub-ventricular zone radial glial cell line (SVG) as normal controls, for the analysis of selective effects on GBM cells. We examined that in 24 h, a dose of 150 μg/ml generated differentiated phenotype of SVGs and at a lower dose of 100 μg/ml no major morphological changes were observed, although a few phase lucent vacuoles were initially observed that disappeared with time (Figure 4A, also see normal F-actin organization in SVG inset). In the same time span of 24 h of drug treatment (100 μg–150 μg/ml, re-supplemented per 12 h), LN229 GBM cell lines showed cell contraction and membrane ruffling (Figure 4A, see red arrows*, please also see* Supplementary Figure S5A for enlarged images of membrane ruffles), cell swelling (Figure 4A, see cyan arrows), cell rounding, de-adhesion and cell lysis (Figure 4A, see magenta arrows). The F-actin organization (cyan, see inset in 100 μg/ml panel) was majorly disturbed with a total loss of actin cables.

Time course monitoring of the cytotoxicity event brought forth an interesting observation that by about 12 h of treatment, the tumor cells were filled with phase lucent vacuoles (*see top insets* in LN229 panel) that progressively fused to generate large vesicles and invariably occupied the entire cell space. These phase lucent vacuoles were identified to be macropinosomes *via* the dextran-TMR uptake assay (please see Supplementary Figure S5B). The delivery of dextran TMR to the cytoplasm and associated cell rounding as shown in middle inset of Figure 4A suggest that the extensive macropinocytotic fluid intake and its delivery to cytoplasm may have generated tremendous hydrostatic pressure on the plasma membrane and internal organelles resulting in cell swelling and squashed nucleus (*as seen in the inset*).

The live/dead and cell permeability assay (Calcein-AM/EtBr based) showed almost complete death of LN229 GBM cells by about 48 h of BM treatment but SVG and human dermal keratinocytes (HaCaT) normal controls remained unaffected at a dose of 100–150 μg/ml (Figures 4B,C).

In order to probe the re-emergence of tumor cells from any residual cells, recovery assays were made wherein BM was incubated for 2–5 days and the cultures were followed for another 2 days without compound replenishment. The results showed that even though there was visible complete cell death in 2 days, the extract un-replenished cultures showed 2%–5% recovery when treated for only 2 days, whereas there was no recovery of tumor cell growth, if the cultures were continuously treated for 5 days. This suggests that certain population of cells in GBM tumor cell cultures needed longer treatment to not recover from the BM induced cytotoxic effects (Figure 4).

### *Bacopa monnieri* Whole Plant Extract Causes Catastrophic Physico-Chemical Changes in the GBM Tumor Cells

To understand the differential catastrophic phenotype (Figure 4A) acquired by the glioblastoma cells *vs.* the normal cells, we examined the possible molecular changes that can cause excessive macropinocytosis dependent hydrostatic stress.

By standard Nomarski (DIC) optics and phase contrast live cell imaging, we had observed extensive cell swelling and substrate de-adhesion in BM treated tumor cells (Figure 4A). Hence, we speculated high intracellular calcium driven fluid uptake in LN229 GBM cells (Falcone et al., 2006; Kabayama et al., 2009) which was indeed aptly supported by the data on calcium imaging (Figure 5A). BM treated tumor cells showed approximately 3–3.5 fold increase in intracellular calcium levels at the dosages of 100 μg/ml to 150 μg/ml, respectively in comparison to BM untreated controls.

Increased cellular calcium is known to positively feedback CaMK2A transcription, which was indeed noticed in the real-time PCR assay in LN229 treated *vs.* untreated cells (Figure 5B). It is to be noted that this assay was made with a treatment dose of 100 μg/ml BM because at a higher concentration, most of the cells had lysed and the amount of RNA recovered was low and of poor quality.

Even though the LN229 cells showed significantly elevated cellular calcium, which is directly related to generation of oxidative stress (Görlach et al., 2015), we did not find a concomitant increase in anti-oxidant SOD1 enzyme by the cancer cells, which may have competitively enabled survival and reduced cell damage. However, it was also ascertained that the cell lysis caused due to tremendous cell swelling was not due to necroptosis pathway as there was no increase in RIPK1 and RIPK3 transcripts, crucially involved in this process (Figure 5B; Jose et al., 2014).

Western blot studies supported the real time data in which both non-phospho CaMK2A and its phosphorylation at T286 was found to be significantly higher in treated *vs.* untreated glioblastoma cells (Figure 5C). It is to be further noted that in such acute fluid stress, the house keeping genes and several other genes were totally disturbed; therefore, the equal loading control was ascertained with BCA assay.

Since cancer cells are known to influx a lot of calcium through extra-synaptic NMDA receptors upon phosphorylation of NR2B subunits, we examined the involvement of NR2B and its downstream effector phospho-CREB (Li and Hanahan, 2013). Interestingly, we found that NR2B and its downstream effector phosphorylation was rather reduced upon BM treatments (Figure 5C), suggesting that the extracellular calcium intake was not involved in cell swelling.

The data on the large amount of fluid uptake in response to high intracellular calcium and its consequent impact on plasma membrane stretch in BM treatments, was further supported by the observation that the levels of phosphorylated caveolin protein was enhanced in BM treated tumor cells whereas total cellular pool was either unchanged (100 μg/ml) or reduced (150 μg/ml; Figure 5D). CAV1 protein, is a marker of surface structures called the caveolae which reserves excess membranes in its folds to enable expansion of membrane in the event of stretch stimulus intercepted by the cells (Hayer et al., 2010; Sinha et al., 2011; Parton and del Pozo, 2013; Echarri and Del Pozo, 2015). Extensive stretch forces, however, deplete the cells of its caveolar reserves, which either become flattened and therefore are lost from the surface or are pinched in before flattening into the endocytic vesicles. CAV1 in flattened areas is phosphorylated at Y14 which destabilizes CAV1 pool from the plasma membrane and augments membrane tension (Joshi et al., 2012; Zimnicka et al., 2016). Observed increase in Y14 phosphorylated pool of CAV1, in BM treatments, implicated disassembly of caveolae (Figure 5D), suggesting that the BM treatment must have generated tremendous membrane stretch leading to the loss of surface tension buffering caveolar reserves. To confirm these observations, we performed sucrose density gradient centrifugation to separate raft/caveolae from non-rafts components of the cell (Figure 5E). Dot blot based estimation of the levels of surface raft/caveolar caveolin *vs.* non-raft/non-caveolar caveolin (fractions with non-raft marker protein K-RAS), showed that with BM treatment less CAV1 protein was identified in the raft fraction, suggestive of loss of caveolar caveolin (Figure 5E; Hancock, 2003; Ariotti et al., 2014). It is known that upon extensive hypo-osmotic membrane stretch stress, the caveolae flatten to provide excess membrane reservoirs and the caveolin-1 protein disperses on the surface and becomes more mobile in non-raft components before it is phosphorylated and endocytosed (Parton and del Pozo, 2013). Hence, the loss of caveolin-1 signal from the detergent resistant fractions (raft) and mixing of the detergent fractions with the markers of the non-detergent resistant fractions (KRAS) provided a good support to the observation that the LN229 cells had undergone acute biomechanical challenge and mechanical stretch by BM treatment which further led to irreversible membrane permeability and membrane lysis (assayed through enhanced permeability to normally cell impermeable EtBr (Figures 4B,C). Also, since caveolar caveolin is known to dampen intracellular calcium, removal of caveolin from the surface fraction and its phosphorylation could have also contributed to enhanced intracellular calcium build up which may have consequently induced high fluid uptake and cell swelling (Yeh et al., 2014).

### Bacoside A Presents Selective Chemical Features Governing *Bacopa monnieri* Anti-Tumorigenic Efficacy

We examined the structure-function relationship of the “different components” of the BM plant extract (Figure 6A; Supplementary Figure S6) through docking and simulation studies of CaMK2A enzyme (amino acid sequence from 200–310 was included which has the major 286–289 amino acid phosphorylation site, catalytic domain and association domain; Hudmon and Schulman, 2002). Our data on the fidelity of ligand binding (represented by the glide score) showed that Bacoside A, which is the major bioactive component of BM, had the highest GlideScore in the T-site (Figure 6A), suggestive of high affinity binding, which was followed by Bacopasaponin C and Bacopaside I. Further, docking and 50 ns simulation of Bacoside A binding to CaMK2A showed that Bacoside can sterically fit well in the enzyme pocket (Figures 6B,D) and its two sugar moieties, glucose and arabinose formed hydrogen bonds with the protein at two stereo centers Glu216 (in the catalytic domain) and Thr310 (in the association domain) respectively. For H bonding also see Supplementary Figure S7 which shows interaction of Bacoside A arabinose sugar interaction with Glu216 and Thr310 of CaMK2A and Bacoside A glucose sugar moiety with Thr310. For other BM component docked models see Supplementary Figure S6. These results again support the hypothesis that CaMK2A must be a direct target of BM and its component Bacoside A. By binding to CaMK2A enzyme, Bacoside A would have induced its phosphorylation and subsequently enabled its binding to ER membrane ryanidone receptors to induce excessive calcium release, causing extensive fluid uptake by macropinocytosis, consequently swelling the cell and forcing lysis.

### Bacoside A Is Sufficient to Attenuate Tumor Growth via Induction of Catastrophic Macropinocytotic /Vacuolization Stress

**Figure 7:**
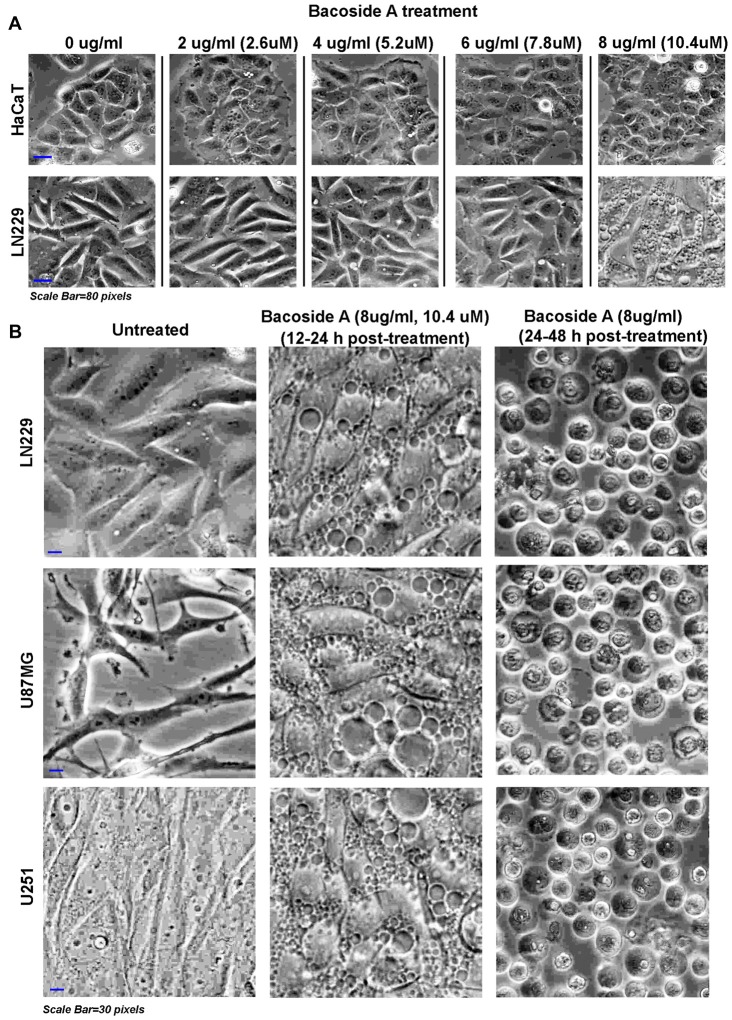
Dose response of Bacoside A demonstrates extensive vacuolization resulting in cell swelling and glioblastoma tumor cell death. **(A)** shows phase contrast images of confluent cultures of HaCaT (normal control cells) and LN229 glioblastoma cell line that were treated with varying doses of Bacoside A for 12 h. A dose of 8 μg/ml and above showed appearance of cytoplasmic vacuoles only in tumor cells and these vacuoles progressively fused with time to occupy the entire cellular space. **(B)** Different glioblastoma cell lines were used to confirm the consistency of Bacoside A vacuolization induced cytotoxic effects at a dose of 8 μg/ml at various time points. Bacoside A treatments produced phase lucent vacuoles, characteristic of macropinosomes, that progressively occupied the entire space, and caused cellular hypertrophy by 12–24 h of treatment (**B**, middle panel) and beyond this time period, the cells were seen to transform into fluid filled, swelled, rounded structures with squashed intracellular organelles and nuclei and many showed necrotic phenotypes (**B**, last panel). At least five random fields per time point were imaged and analyzed. The images are representative of five independent experiments.

Based on our results, Bacoside A induced phospho-CaMK2A dynamics which appeared to be majorly responsible for GBM cell lysed phenotype. Hence, we titrated the effective dose-time response for Bacoside A in human normal keratinocytes (HaCaT) and LN229 glioblastoma cells (Figure 7A) and determined 8 μg/ml to be the efficacious dose for further assays as it generated similar vacuolization effects as observed in BM treatment but did not cause any change in the phenotype of normal controls (Figure 7A). In order to further determine the consistency of Bacoside A vacuolization induced cytotoxic effects at a dose of 8 μg/ml, we followed the treatments in other glioblastoma cells which carry different mutations. In all the cell lines tested, i.e., LN229/U87MG/U251, Bacoside A treatments produced phase lucent vacuoles, characteristic of macropinosomes, which progressively fused into larger vacuoles and occupied the entire space, causing cellular hypertrophy by 12–24 h of treatment (Figure 7B, middle panel) and beyond this time period, the cells were seen to transform into fluid filled, swelled, rounded structures with squashed intracellular organelles/nuclei and many showed necrotic phenotypes (Figure 7B, last panel). It is to be noted that unusually large fluid filled vesicles in the entire cellular space is bound to exert tremendous hydrostatic stress on the intracellular organelles and on the plasma membrane even before they actually release the stored fluid volume into the cytoplasmic space.

**Figure 8:**
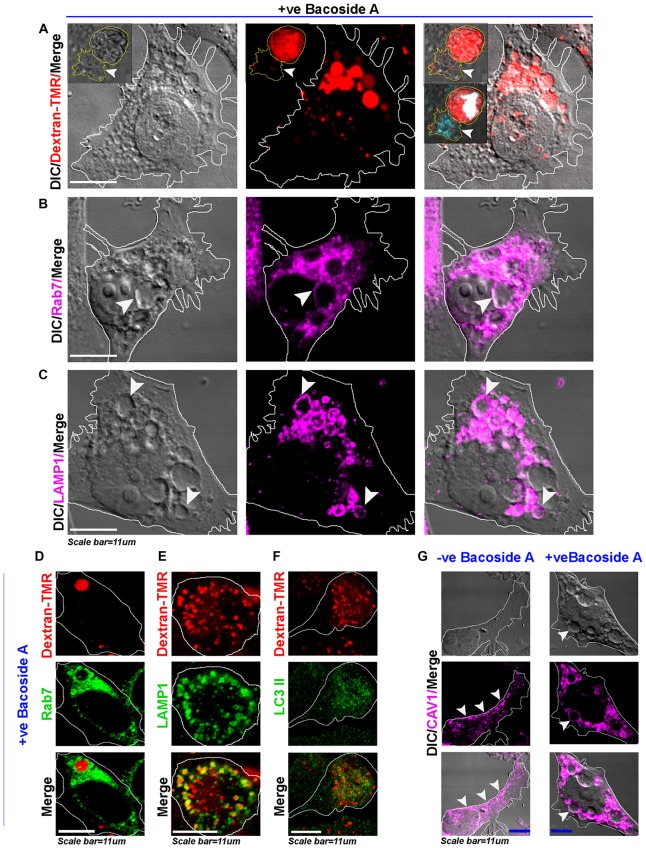
Bacoside A induced enlarged vacuoles are macropinosomes that undergo time dependent pre-lysosomal fusion. **(A)** Confocal images of Bacoside A (12 h) treated LN229 glioblastoma cells showed large vacuoles positive for dextran-TMR (red), a marker of macropinosomes. Insets in the panel shows that in 24 h Bacoside A treated LN229, cells had rounded off and were filled with dextran-TMR, delivered by macropinosomes to the cytoplasm. Hence, cell swelling was a net consequence of excess fluid uptake by the tumor cells. The white arrowhead points to a necrotic cell attached to swelled cell with squashed nucleus (cyan/white). The vacuoles were identified to be positive for late endosome/macropinosome markers. **(B)** Rab7 and **(C)** LAMP1. White arrows in panels **(B,C)**, points to the localization of Rab7 and LAMP1 surface proteins to the membranes of vacuoles. **(D)** shows Dextran-TMR positive macropinosome surface positivity for Rab7 (green). Similarly, **(E)** shows Dextran-TMR positive macropinosome surface positivity for LAMP1 (green) but **(F)** not for LC3II, an autophagosome marker (also see Supplementary Figure S8). **(G)** The Bacoside A treated LN229 tumor cell vacuolar membranes were also positive for caveolin-1 protein and in many vesicles it was noticed in the vesicle lumen, suggestive of both caveolar/surface origin of vacuoles and non-caveolar surface origins from the caveolar flattened areas respectively. Overall, treated cells showed major loss of surface CAV1 in comparison to untreated LN229 cells. The images are representative of five independent experiments.

As rapid intake of extracellular fluid is a hallmark of macropinocytosis, we found that the tracer dextran-TMR (a fluid phase fluorescently labeled sugar) was taken up by all the cells within 10–30 min with an appearance in the vacuolar structures and was also observed in enlarged vacuoles formed by homotypic fusion over time (Figure 8A). However, the untreated cells showed much fewer macropinosomes, which were considerably smaller in size in comparison to the treated cells, as also observed in the case of BM treatments (Supplementary Figure S5B). Also, in 24 h post treatment, the swelled and rounded cells were observed to be completely filled with the dextran-TMR and cells showed squashed nuclei, whereas several cells had already turned necrotic by this time (*see insets in* Figure 8A top panel). As intracellular itineraries of macropinosomes significantly overlaps with endocytic pathways (Overmeyer et al., 2011; Kitambi et al., 2014; Ha et al., 2016; Lima et al., 2017), the macropinocytotic vacuoles were also evidenced to recruit late endosomal-lysosomal markers Rab7 and LAMP1 (Figures 8B–E) on their membranes but did not show LC3II positivity (Supplementary Figures S9A–C; Figure 8F assessed by Dextran-TMR and LC3II immunofluorescence based co-labeling), suggesting that vacuoles were not autophagosomes and that the macropinosomes were stalled at pre-lysosomal phase. Further evidence of the recruitment of caveolin-1, a major component of surface membrane tension buffering structures; to the vacuolar membranes (Figure 8G) suggest rapid internalization of surface membranes in treated *vs.* untreated tumor cells leading to a major loss of plasma membrane and increase in surface tension which may aid in membrane fragility and cell lysis.

### Bacoside A Activates Extensive T286 Phosphorylation of CaMK2A and Causes Non-Apoptotic Glioblastoma Cell Death

**Figure 9:**
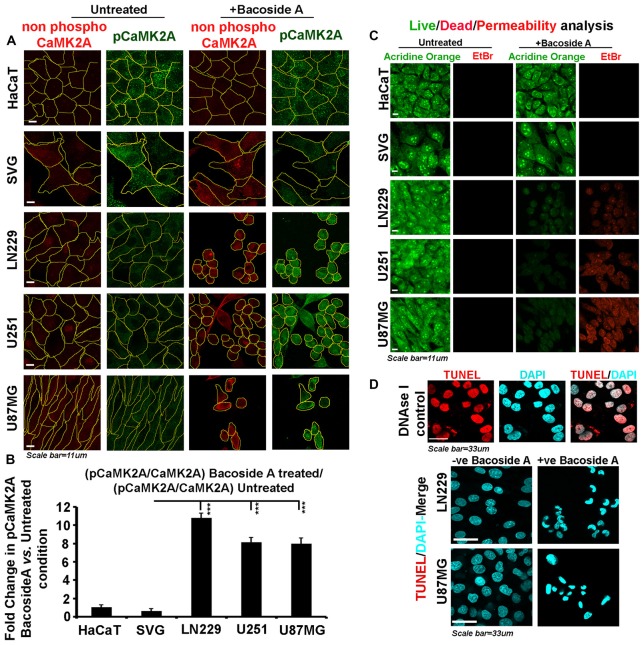
Bacoside A activates extensive T286 phosphorylation of CaMK2A and non-apoptotic glioblastoma cell death. **(A)** Single cell quantitative image analysis procedure (see John et al., 2017) was employed to measure non-phospho CaMK2A and phospho CAMK2A T286 protein levels in normal human cells: SVG-subventricular zone derived glial cells; HaCaT-epidermal keratinocytes and in various GBM patient derived tumor cell lines: U87MG, U251 and LN229 upon Bacoside A (8 μg/ml) treatment for 24 h. **(B)** The proportion of phosphorylated CaMK2A determined by normalizing average signal of pCaMK2A-T286 to average signal from non-phospho CaMK2A in BM treated cells *vs.* the ratio in the normal cells, showed higher levels of pCaMK2A in treated tumor cells but not the normal control cell lines. Please note that just “CaMK2A” in the formula refers to predominantly the non-phosphorylated pool. The analysis is representative of three independent experiments and in each experiment protein signal was quantified from over 200 cells in five random fields. **(C)** Acridine orange (green, live cell marker probe)/EtBr (red, damaged and dead cell marker dye) based cytotoxicity and viability analysis over 24 h post Bacoside A treatment at 8 μg/ml showed specific cytotoxic effects on LN229/U87MG/U251 glioblastoma cell lines *vs.* the normal cells. **(D)** LN229 and U87MG GC treated with 8 μg/ml of Bacoside A for 48 h were negative for TUNEL staining (indicator assay for apoptotic cell death). ***p < 0.001. Error bar = SD The analysis is representative of three independent experiments.

In order to confirm that akin to BM treatment, Bacoside A itself can extensively phosphorylate the CaMK2A pool in various glioblastoma cells with different genetic landscapes, we treated the normal human cells: SVG-subventricular zone derived glial cells; HaCaT-epidermal keratinocytes and various GBM patient derived tumor cell lines: U87MG, U251 and LN229 with Bacoside A (8 μg/ml) treatment for 24 h (Figure 9A). Immunostaining and single cell based image quantitation of the proportion of CaMK2A phosphorylation in treated *vs.* untreated cells in glioblastoma cells and normal cells clearly suggested significant increase in phospho-CaMK2A in all glioblastoma cell lines vs. their untreated controls (Figures 9A,B).

We next examined whether Bacoside A generated selective membrane permeability defects in glioblastoma cells vs. the normal cells and we indeed noticed an extensive signal of EtBr in treated GBM cells (Figure 9C) confirming that major BM cell lysis effects were a function of the action of Bacoside A. Our observations on loss of membrane integrity to be the final event in Bacoside A mediated necrotic like death was further confirmed by the lack of apoptosis associated DNA fragmentation as assessed by the TUNEL assay (Figure 9D).

### Bacoside A Uncontrolled Macropinocytotic Effects Are Dependent on Phospho- CaMK2A Mediated Intracellular Calcium Upsurge Leading to Cytoskeletal Damage and Enhanced Membrane Permeability Associated Cell Lysis

To delve deeper into the mechanism and confirm the dependency on “CaMK2A phosphorylation” for the major phenotypic effects produced by Bacoside A, we ran several inhibitor based controls.

Canonical mode of CaMK2A phosphorylation requires increase in calcium and calcium-calmodulin complex to bind at the catalytic domain and this calcium can be provided by extracellular influx through NMDA channels. Phosphorylation of NR2B subunit is an important event in this process and Thr286 amino acid unit of CaMK2A is a major site of phosphorylation (Kitambi et al., 2014). Non-canonical modes of phosphorylation of CaMK2A have been described, wherein low/below basal/basal levels of calcium-calmodulin were sufficient for phosphorylation (at Thr286 and Ser135) and the phosphorylation was majorly mediated by Coenzyme A (McCoy et al., 2013). However, independent of the modes of activation, CaMK2A is a major cellular calcium sensor and phosphorylation state of this enzyme is crucially involved in the release of intracellular calcium from the ryanidone channels of the ER. Hence, we wanted to understand whether Bacoside A required the following to phosphorylate and activate CaMK2A: (i) extracellular calcium flux; (ii) increase in intracellular calcium; and (iii) requirement of calmodulin. We wanted to further examine whether the Bacoside A mediated CaMK2A extensive phosphorylation was responsible for intracellular calcium increase and also whether the extensive and catastrophic macropinocytosis was a stimulus dependent function of the net rise in intracellular calcium levels.

**Figure 10:**
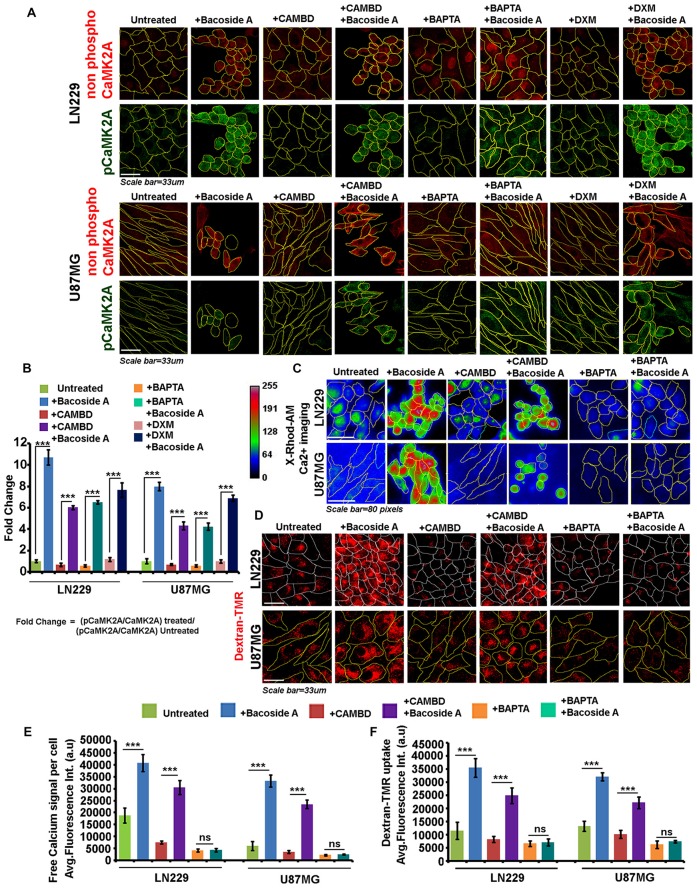
Bacoside A uncontrolled macropinocytotic effects are dependent on phospho-CAMK2A mediated intracellular calcium upsurge. **(A)** Single cell quantitative image analysis procedure (see John et al., 2017) was employed to measure non-phospho CaMK2A and pCaMK2A-T286 protein levels in various GBM patient derived tumor cell lines: U87MG and LN229 upon Bacoside A (8 μg/ml) and inhibitor treatments for 24 h. **(A,B)** The proportion of phosphorylated CaMK2A was determined by normalizing average signal from pCaMK2A-T286 to average signal from non-phospho CaMK2A in treated cells. Results showed higher levels of pCaMK2A in Bacoside A treated tumor cells *vs.* the untreated control cell lines. Please note that just “CaMK2A” in the formula refers to predominantly the non-phosphorylated pool. Similar results were obtained even in presence of CAMBD (competitive inhibitor of CaMK2A phosphorylation), DXM (Indirect inhibitor of CaMK2A phosphorylation, inhibits NMDA dependent calcium influx) and BAPTA (a chelator of intracellular calcium), indicating that Bacoside A mediated CaMK2A phosphorylation activation were not majorly dependent on intracellular calcium or calmodulin levels. **(C)** Live cell X-Rhod1-AM based imaging of intracellular calcium release, free calcium flux, upon BM treatment was performed. **(C,E)** Results showed significantly increased levels of free calcium in Bacoside A treated *vs.* untreated LN229/U87MG GC even in the presence of CAMBD but not when BAPTA, an intracellular calcium chelator was used, indicating that Bacoside A releases intracellular calcium. **(D,F)** Dextran-TMR fluorescence quantitation to measure extent of macropinocytosis, indicated that Bacoside A treatment significantly enhanced macropinocytosis even in the presence of CAMBD but not when BAPTA, a calcium chelator was used, indicating that Bacoside A macropinocytosis induction was based on its effects on intracellular calcium levels. It is to be noted that calcium is one of a known inducer of macropinocytosis. The analysis is representative of three independent experiments. ***p < 0.001, Error bar = SD.

To address these three crucial questions, the LN229 and U87MG glioblastoma cells were pre-treated separately with: (1) Dextromethorphan (DXM, 10 μM) to inhibit NR2B receptor phosphorylation which will prevent extracellular calcium influx (Marquard et al., 2015); (2) Calmodulin binding domain peptide (CAMBD, 200 nM) that sequesters calmodulin cellular pool and also acts as a pseudo-substrate and suppress CaMK2A phosphorylation (McCoy et al., 2013); and (3) BAPTA (20 μM) to chelate intracellular calcium which would inhibit CaMK2A phosphorylation (Figure 10A). Following the inhibitor treatments/preloading, the cells were treated with Bacoside A with the respective inhibitors and the following parameters were assessed: (1) levels of phosphorylation of CaMK2A at T286 site immunofluorescence based image quantitation; (2) levels of intracellular calcium-XRhod1-AM assay; (3) extent of macropinocytosis-Dextran TMR assay; (4) extent of cytoskeletal damage-Rhodamine Phalloidin staining and finally; and (5) loss of cell permeability and viability-acridine orange/EtBr assay. These assays were expected to enable our understanding on the Bacoside A dependent CaMK2A phosphorylation driven calcium upsurge as an indispensible mechanism for catastrophic macropinocytosis induced cell lysis. It is to be noted that these inhibitors have been previously shown to generate very low and below basal levels of calcium and calmodulin but cannot completely sequester it (McCoy et al., 2013).

**Figure 11:**
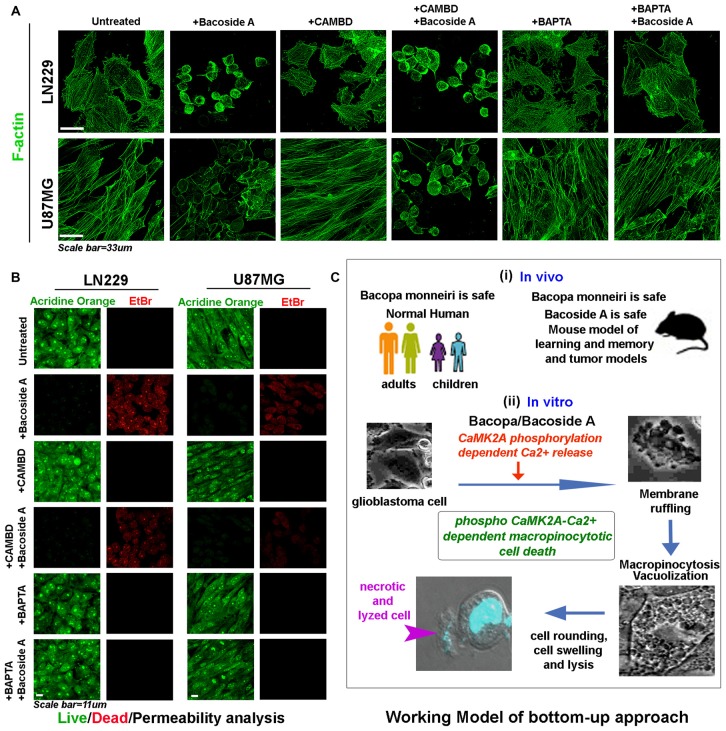
Bacoside A mediated cell swelling and hydrostatic pressure changes results in cytoskeletal damage and increase in membrane permeability associated cell death. **(A)** Both LN229 and U87MG glioblastoma cell lines showed significant loss of cytoskeleton integrity upon Bacoside A treatment for 24–48 h, even in the presence of CAMBD but not when BAPTA, a calcium chelator was used, indicating that Bacoside A cytoskeletal damaging effects were dependent on intracellular calcium levels. As shown in Figure 10D, chelation of calcium dramatically reduces macropinocytosis and cell swelling, hence calcium induced excessive cell fluid uptake driven hydrostatic pressure in cells, ruptures the actin cables. **(B)** Acridine orange (green, live cell marker probe)/EtBr (red, damaged and dead cell marker dye) based cytotoxicity and viability analysis post 24 h Bacoside A treatment at 8 μg/ml showed specific cytotoxic effects on LN229/U87MG/U251 glioblastoma cell lines even in presence of CAMBD but not when BAPTA, a calcium chelator was used, indicating that Bacoside A cytotoxic effects were dependent on intracellular calcium driven excessive fluid uptake and consequent build up of intense hydrostatic pressure that ruptures the plasma membrane. The analysis is representative of three independent experiments. **(C)** The conclusive working model of the study highlights a “bottom up approach”, wherein, BM, the anti-GBM potential target drug, is already demonstrated to be safe in both adult and children and its active component is additionally shown to be safe in normal mice and in tumor models. A further mechanism based *in vitro* study highlights a novel mechanism by which both Bacopa and Bacoside A may majorly work to enable GBM specific cytotoxicity.

As clearly depicted by the results, NR2B receptor driven calcium intake was not required for Bacoside A CaMK2A phosphorylation (Figure 10A, last panel; Figure 10B). Inhibition of calmodulin binding to CaMK2A, should normally prevent this kinase structural change which is essential for phosphorylation at T-site of the catalytic domain and should therefore decrease the release of intracellular calcium. We found this to be true when CAMBD treatment was given alone to the tumor cells (Figures 10A,B). Surprisingly however, when CAMBD pre-treated cells were further treated with Bacoside A (along with CAMBD), CaMK2A phosphorylation did occur, albeit a little less in comparison to Bacoside A treatment alone. BAPTA treatment that restricted/chelated intracellular calcium also could not inhibit CaMK2A phosphorylation in Bacoside A treatment (Figures 10A,B). Hence, we find that Bacoside A in presence of very low/below basal or basal levels of calcium and calmodulin could cause CaMK2A conformational change that allows this kinase to be phosphorylated at its major site T-286-287. An associated follow up on the Bacoside A effects on calcium release and macropinocytosis in the presence or absence of inhibitors suggested that Bacoside A treatment generated high intracellular calcium flux and macropinocytosis which was also appreciably co-related in CAMBD + Bacoside A treated cells but BAPTA alone and BAPTA + Bacoside A treatments showed diminished intracellular calcium and reduced macropinocytosis (Figures 10C–F). This suggests that even though increase in calcium was not required for Bacoside A dependent CaMK2A phosphorylation, availability of the Bacoside A mediated intracellular calcium release was required for enhanced macropinocytotic effects. The inhibitor + Bacoside A treatment results on changes in intracellular calcium levels and macropinocytosis corroborated well with the extent of cytoskeletal damage (Figure 11A) and compromise in membrane permeability (Figure 11B) leading to cell lysis. The BAPTA mediated sequestration of intracellular calcium release, rescued cytoskeleton and membrane integrity which was severely compromised in Bacoside A or Bacoside A + CAMBD conditions.

Therefore, Bacoside A alone could exert BMs anti-tumorigenic effects, however we cannot deny that phosphorylation of other amino acids on CaMK2A as well as phosphorylation of other CaMK2A isoforms by Bacoside may also be involved to enhance the effect. Since CaMK2A is also expressed in several other tumors, we anticipate a similar mechanism of action of Bacoside A to function in the eradication of various other tumor niches as well.

As highly aggressive tumor are now evidenced to develop differential extracellular pH gradients which is linked to tumor heterogeneity and chemoresistance (Supplementary Figure S8A; John et al., 2017), we find that tumor pH gradients were unable to compromise Bacoside A vacuolization induced cytotoxic effects (Supplementary Figures S8B–F). Hence, Bacoside A presents a good potential to act as a new treatment against cancers of various origins as is also diagrammatically depicted in the working model shown in Figure 11C.

## Discussion

The major objective of this study was to identify and test the efficacy of a natural compound that can specifically drive tumor cell death* via* hijacking the delicate biomechanical balance of glioblastoma tumor cells, akin to the action of synthetic product vaquinol-1, that has shown a good potential as a new drug against GBM but unfortunately was found to be associated with non-specific toxicity on extended dosages (Gilbertson, 2014; Kitambi et al., 2014).

The rapidly expanding GBM tumor heterogeneity and recurrence, chemo and radio-resistance, oncogenic mutations, alternative splice variants, gene polymorphism and rapid chromatin remodeling has posed enormous difficulties in identifying the specific cellular targets, hence a “target based” approach coupled with phenotypic screens that alter the physico-chemical characteristics of cancer cells, is now being revisited in anti-cancer drug discovery efforts (Kitambi et al., 2014; Maltese and Overmeyer, 2014; Patil et al., 2014). Vacquinol-1 was recently identified to be a potential new anti-GBM drug *via* this approach. The authors generated primary cell lines from GBM patients and tested the cytotoxicity of the synthetic compound by counter screening in mouse ES cells, human fibroblasts cultures and orthotopic model of GBM in zebrafish (Kitambi et al., 2014). This report brought forth insights into the existing vulnerability of glioblastoma cells in the physico-chemical realm of tumor cells. The biomechanics of macropinocytosis or bulk phase endocytosis is extensively exploited by cancer cells to drive excess nutrient from the extracellular microenvironment (Ha et al., 2016). However, induction of massive macropinocytosis can lead to cell death due to extensive hydrostatic pressure (of fluid intake) on the plasma membrane and internal organelles resulting in membrane deformation, cell swelling and rupture (Overmeyer et al., 2011; Kitambi et al., 2014; Maltese and Overmeyer, 2014). The obstacle in the escalation of Vacquinol-1 into clinics was identified as unrelated toxicity at an effective therapeutic dose, upon prolonged administration (Overmeyer et al., 2011; Kitambi et al., 2014).

Such kind of *de novo* drug development approach is also expensive and time consuming; hence the knowledge gained by some crucial efforts in this direction can be channelized into screening for the natural molecules that may exert the same mode of action. This bottom up approach essentially repurposes the drug known for its, safety, efficacy and bioavailability across different systems of medicines (including alternative medicinal systems such as the Ayurveda). Many tumor medications are now being inspired by natural phytochemicals, however, characterizing the mechanisms of action of these compounds and molecules is essential for their development and usage as new anti-cancer drugs.

In order to “alternatively” drive GBM cell uncontrolled macropinocytosis, we carefully studied the literature associated with the process of macropinocytosis and cancer. Besides Vacquinol-1, a chalcone related compounds such as MIPP was shown to generate the methuosis effects, however it showed cytotoxicity on normal cells as well (Overmeyer et al., 2011). Sphingosine kinase inhibitors such as SK1-I and FTY720 also generated macropinocytotic pre-lysosomal accumulation of dilated vesicles but cell death was not reported (Lima et al., 2017). β-lapachone and AS1411 anti-cancer aptamers were also found to generate high vacuolization effects through independent mechanisms (Ma et al., 2015; Reyes-Reyes et al., 2015).

Calcium has been shown to promote macropinocytosis (Falcone et al., 2006). However, whether increase in intracellular calcium can promote excessive macropinocytosis was not clearly studied. Although recently non-canonical Wnt mediated increase in intracellular calcium has been reported to cause glioma stem-like cells death (Bhuvanalakshmi et al., 2015). Tumor cells express a crucial calcium sensing and intracellular calcium releasing protein CaMKII (Wang et al., 2015). The CaMKII or CaMK2 is a calcium sensing serine/threonine (S/T) protein kinase with four isoforms 2A, 2B, 2D and 2G (also denoted as α, β, δ, γ) which are implicated in various processes associated with tumor progression.

The TCGA data based information available on the expression of CaMKII in gliomas and the fact that its high phosphorylation can mediate intracellular calcium release, motivated us to examine whether excessive activation of CaMKII can increase cellular calcium to induce excessive macropinocytosis thereby generating hydrostatic stress and cell death in GBM cells. BM plant extract and its bioactive component Bacoside A are documented to enhance CaMK2A phosphorylation in the process associated with learning and memory.

BM, an Indian traditional medicine, is cost-effectively available as a herbal ayurvedic “off the counter” medicine throughout the world and is sold under various brand names (e.g., under the name “Brahmi” from Himalaya Herbals, Aryavaidyashala-Kottakal, Jiva Ayurveda etc.). Interestingly, the pharmacokinetics, biosafety, oral bioavailability (or by nano-drug carriers; Jose et al., 2014), efficacy and stability of our compound of interest is extensively tested in both mouse, rat and humans and this compound is considered safe for even normal human intake due to its well proven role in enhancing learning and memory processes both in children and adults (Nathan et al., 2001; Janani et al., 2010; Morgan and Stevens, 2010; Prakash et al., 2011; Lisman et al., 2012; Nandagaon and Kulkarni, 2013; Patil et al., 2014; Mallick et al., 2015). Bacopa has anti-epileptogenic effects, hence may be further useful in managing glioma associated epileptic seizures (Maschio, 2012; Yadav and Reddy, 2013).

We used established GBM cell lines, to test Bacopa effects as a CaMK2A phosphorylation inducer, due to the fidelity of expression of target protein of interest (CaMK2A) and the expression patterns corroborated well in GBM patient tissues (Figures 1–3).

The cytotoxicity of BM whole plant extract and Bacoside A towards normal cell line, in our experiments, were identified to be insignificant at a dose 100 μg/ml and 6–8 μg/ml, respectively (Patil et al., 2014; Mallick et al., 2015). However, these dosages were effective in killing the GBM tumor cells. According to standard clinical trials, this determined “*in vitro*” dosage would escalate to 100–200 mg (whole plant extract) and 6–8 mg (Bacoside A) respectively per day, to be administered for 3–6 months in GBM mouse model. The mouse and human subjects are found to be tolerant to much higher doses of this compound, which is nearly 300–350 mg per day for 3–6 months, respectively (Nathan et al., 2001; Morgan and Stevens, 2010; Majumdar et al., 2013; Yadav and Reddy, 2013). Bacoside A has also been shown to be anti-tumorigenic in breast, prostate and lung tumor cultures and in animal models (Janani et al., 2010; Prakash et al., 2011; Patil et al., 2014; Mallick et al., 2015), however its mechanisms of action involving differential and specific tumor cell death *vs.* normal cells had remained unclear. Our mechanism-based study suggests that BM or Bacoside A can be safely administered in the suggested concentrations for GBM specific cytotoxicity. BM has certain other advantages over Bacoside A in that it has betulinic acid that acts as an anti-glucosidase and prevents cleavage of carbohydrate moieties on the Bacoside, hence stabilize its activity (Pisha et al., 1995). However, as betulinic acid is also available in purified form, it can be co-administered with Bacoside A *in lieu* of BM (we plan to test this in the orthotopic model of GBM). The biological processes involved in BM/Bacoside driven cell death included: (1) extensive phosphorylation of CaMK2A; (2) phosphorylated CaMK2A served to increase the cytoplasmic pool of calcium *via* its extrusion from the ryanidone channels of ER; (3) high calcium enhanced the rate and extent of macropinocytosis; (4) macropinocytotic vesicles depleted the plasma membrane of it excess membrane reserves-the caveolae, hence enhanced membrane tension and vulnerability to leakage; (5) the macropinocytotic vesicles fused, enlarged and occupied the entire cell space, generating cellular congestion and hypertrophy; (6) the enlarged macropinosomes and the fluid released from them to the cytoplasmic space, caused cell swelling and rounding, suggesting that excessive fluid generated enormous hydrostatic stress on the plasma membrane hence causing cytoskeletal damage and membrane rupture of the fluid filled and swelled tumor cells; and (7) the tumor cells eventually leaked their content, shrank and lost viability *via* necrosis like cell death. Standard DIC images showed evidences of membrane ruffling, cell contraction, cell rounding, high disintegrity of intracellular organelles and cell lysis in the treated cells (Supplementary Figure S5, Figures 4, 7, 8, 11). Interestingly, these evidences solidly corroborated with macropinocytosis and vacuolization events observed upon Vacquinol-1 administration (Kitambi et al., 2014). The mechanism of cell death was essentially identified to be a combination of cell swelling and subsequent levitation induced cell death, “anoikis”, followed by necrosis (Figure 7; Taddei et al., 2012; Pasparakis and Vandenabeele, 2015) as we did not find any significant increase in necroptosis marker RIPK1 and RIPK3 or signs of apoptotic DNA fragmentation (Figures 5B, 9D).

While it is well known that caveolar structures buffer mechanical stretch of plasma membrane during hypo-osmotic plasma membrane stretch (fluid movement into the cell), the excessive cell swelling due to this physical stress depletes cells of caveolar membrane reserves and the cells rounds off to alternatively acquire a least membrane tension geometry (Hayer et al., 2010; Sinha et al., 2011; Parton and del Pozo, 2013; Echarri and Del Pozo, 2015; Zimnicka et al., 2016). The loss of actin cables and initial membrane ruffling in BM/BacosideA treated cells suggested localized points of actin disassembly/depolymerization from the membrane for the release of hydrostatic pressure which was built-up due to the excess fluid intake (Figure 11A).

The normal cells were rather spared from this effect at a similar dose, probably due to adaptation of normal cells to higher pools of phospho-CaMK2A and the regulation of its phosphorylation by protein phosphatases in physiological situation *vs.* in glioma cells, and also probably due to the higher density of resident caveolae in normal cells (Sinha et al., 2011; Parton and del Pozo, 2013; Yeh et al., 2014; Echarri and Del Pozo, 2015; Zimnicka et al., 2016). Some other alternative mechanisms by which raised calcium can be buffered in normal cells can be through enhanced translation of calcium binding/sequestering proteins (calmodulin, parvalbumin etc.) or *via* the tightly regulated dephosphorylation of CaMK2A as mentioned above, however this requires further investigation. Interestingly, normal SVGs (which are the neural progenitor cells) showed differentiated morphology at a dose of about 150 μg/ml BM, but not at 100 μg/ml, which warrants examination of the role of Bacosides in neural progenitor differentiation that may provide insights into the double benefits of simultaneous anti-cancer and neural regenerative effects of Bacopa/Bacoside A administration in gliomas. Since Bacosides are essentially saponins, or mild detergents, they are capable of creating transient-resealable holes in the plasma membrane to gain cellular entry, hence, may be very effective against use in multi-drug resistant, chemoresistant and radioresistant cancer stem cell population which accounts for major recurrence of tumors after surgery. Besides, being a class of amphipathic triterpenoid saponins, they are also highly absorptive on the surface and can penetrate into the cell through this diffusive property (Yoshida et al., 2008). TCGA data in different types of GBMs showed CaMK2A expression, albeit lower than the normal tissues (Figure 1) and other studies also supports this finding in GBM (Jayaram et al., 2016) suggesting that tumor cells “need” but “keeps low levels” of this kinase in comparison to “normal controls”. We hereby showed that “within gliomas” (not to be confused with comparatively lower levels with respect to normal controls), mere examination of the high transcript levels is not sufficient to connect CaMK2A with tumor progressivity. Levels of CaMK2A activated or phosphorylated states have a strong determining impact on tumor cell intracellular calcium levels. Phosphorylated CaMK2A (Thr286) is crucially involved in calcium release from the ryanidone channels of ER. GBM high grade patient tissues showed strict regulation of phospho CaMK2A expression which would prevent cytoplasmic calcium burst that could otherwise adversely affect tumor cell survival.

Positivity of DNA fragmentation assays are demonstrated in some cancer lines upon treatment with BM but the major mechanism of cellular disintegrity in our investigation identified the cell death to be a combination of acute excessive macropinocytotic stress-anoikis-necrosis driven cell death. Further phenotypic screens in several other cancer cells too may confirm CaMK2A enhanced activation by Bacoside A to be useful for treatment of cancers as well as for targeting the quiescent tumor initiating populations, as it functions on the common mechanism of generation of high hydrostatic stress *via* CaMK2A-pCaMK2A-high intracellular calcium-excessive cell drinking pathway (*please see the mechanistic model in* Figure 11C).

Hence, overall the study highlights that: (1) glioblastoma cells are vulnerable to high calcium induced macropinocytotic-hydrostatic stress; (2) BM plant extract/Bacoside A enhances CaMK2A and activates CaMK2A phosphorylation (pCaMK2A) in GCs; and (3) pCaMK2A triggers high calcium release from ER causing macropinocytotic and membrane hydrostatic stress induced tumor cell death. Bacoside A has excellent brain availability through oral routes, hence should be escalated as potential treatment for GBMs. This work further generates fresh insights into the crucial role of biophysical forces as the higher organizing principles in tumor survival and how by hijacking the hub molecules in tumor biomechanical-regulatory circuitries we can generate irreversible force imbalances, thereby eradicating the oncogenic niches.

## Availability of Data and Material

The datasets supporting the conclusions of this article are included within the article and its supplementary files.

## Author Contributions

RM and SJ designed the research; SJ and RM performed the research and wrote the manuscript. KCS performed docking and simulation experiments. All authors have read and approved the final manuscript.

## Conflict of Interest Statement

The authors declare that the research was conducted in the absence of any commercial or financial relationships that could be construed as a potential conflict of interest.

## Footnotes

^1^www.naturalremedy.com

^2^www.himalayawellness.com

^3^https://primerdepot.nci.nih.gov/

^4^www.betastasis.com

## References

[B2] Ariotti N. Fernández-Rojo M. A. Zhou Y. Hill M. M. Rodkey T. L. Inder K. L.. (2014). Caveolae regulate the nanoscale organization of the plasma membrane to remotely control Ras signaling. J. Cell Biol. 204, 777–792. 10.1083/jcb.201307055 24567358 PMC3941050

[B3] Asghar W. El Assal R. Shafiee H. Pitteri S. Paulmurugan R. Demirci U. (2015). Engineering cancer microenvironments for *in vitro* 3-D tumor models. Mater. Today 18, 539–553. 10.1016/j.mattod.2015.05.002 28458612 PMC5407188

[B4] Aspenström P. (2004). Integration of signalling pathways regulated by small GTPases and calcium. Biochim. Biophys. Acta 1742, 51–58. 10.1016/j.bbamcr.2004.09.029 15590055

[B10] Bhuvanalakshmi G. Arfuso F. Millward M. Dharmarajan A. Warrier S. (2015). Secreted frizzled-related protein 4 inhibits glioma stem-like cells by reversing epithelial to mesenchymal transition, inducing apoptosis and decreasing cancer stem cell properties. PLoS One 10:e0127517. 10.1371/journal.pone.0127517 26030909 PMC4452329

[B5] Brocker C. Thompson D. C. Vasiliou V. (2012). The role of hyperosmotic stress in inflammation and disease. Biomol. Concepts 3, 345–364. 10.1515/bmc-2012-0001 22977648 PMC3438915

[B6] Chi M. Evans H. Gilchrist J. Mayhew J. Hoffman A. Pearsall E. A.. (2016). Phosphorylation of calcium/calmodulin-stimulated protein kinase II at T286 enhances invasion and migration of human breast cancer cells. Sci. Rep. 6:33132. 10.1038/srep33132 27605043 PMC5015093

[B7] Echarri A. Del Pozo M. A. (2015). Caveolae—mechanosensitive membrane invaginations linked to actin filaments. J. Cell Sci. 128, 2747–2758. 10.1242/jcs.153940 26159735

[B8] Egami Y. Taguchi T. Maekawa M. Arai H. Araki N. (2014). Small GTPases and phosphoinositides in the regulatory mechanisms of macropinosome formation and maturation. Front. Physiol. 5:374. 10.3389/fphys.2014.00374 25324782 PMC4179697

[B9] Falcone S. Cocucci E. Podini P. Kirchhausen T. Clementi E. Meldolesi J. (2006). Macropinocytosis: regulated coordination of endocytic and exocytic membrane traffic events. J. Cell Sci. 119, 4758–4769. 10.1242/jcs.03238 17077125

[B11] Gilbertson R. J. (2014). Driving glioblastoma to drink. Cell 157, 289–290. 10.1016/j.cell.2014.03.034 24725398

[B12] Görlach A. Bertram K. Hudecova S. Krizanova O. (2015). Calcium and ROS: a mutual interplay. Redox Biol. 6, 260–271. 10.1016/j.redox.2015.08.010 26296072 PMC4556774

[B13] Ha K. D. Bidlingmaier S. M. Liu B. (2016). Macropinocytosis exploitation by cancers and cancer therapeutics. Front. Physiol. 7:381. 10.3389/fphys.2016.00381 27672367 PMC5018483

[B14] Hancock J. F. (2003). Ras proteins: different signals from different locations. Nat. Rev. Mol. Cell Biol. 4, 373–384. 10.1038/nrm1105 12728271

[B15] Hayer A. Stoeber M. Ritz D. Engel S. Meyer H. H. Helenius A. (2010). Caveolin-1 is ubiquitinated and targeted to intralumenal vesicles in endolysosomes for degradation. J. Cell Biol. 191, 615–629. 10.1083/jcb.201003086 21041450 PMC3003328

[B16] Hudmon A. Schulman H. (2002). Neuronal Ca^2+^/calmodulin-dependent protein kinase II: the role of structure and autoregulation in cellular function. Annu. Rev. Biochem. 71, 473–510. 10.1146/annurev.biochem.71.110601.135410 12045104

[B17] Janani P. Sivakumari K. Geetha A. Ravisankar B. Parthasarathy C. (2010). Chemopreventive effect of bacoside A on *N*-nitrosodiethylamine-induced hepatocarcinogenesis in rats. J. Cancer Res. Clin. Oncol. 136, 759–770. 10.1007/s00432-009-0715-0 19916024 PMC11828319

[B18] Jayaram S. Gupta M. K. Raju R. Gautam P. Sirdeshmukh R. (2016). Multi-omics data integration and mapping of altered kinases to pathways reveal gonadotropin hormone signaling in glioblastoma. OMICS 20, 736–746. 10.1089/omi.2016.0142 27930095

[B19] John S. Mishra R. (2016). mRNA transcriptomics of galectins unveils heterogeneous organization in mouse and human brain. Front. Mol. Neurosci. 9:139. 10.3389/fnmol.2016.00139 28018170 PMC5159438

[B20] John S. Sivakumar K. C. Mishra R. (2017). Extracellular proton concentrations impacts LN229 glioblastoma tumor cell fate via differential modulation of surface lipids. Front. Oncol. 7:20. 10.3389/fonc.2017.00020 28299282 PMC5331044

[B21] Jose S. Sowmya S. Cinu T. A. Aleykutty N. A. Thomas S. Souto E. B. (2014). Surface modified PLGA nanoparticles for brain targeting of Bacoside-A. Eur. J. Pharm. Sci. 63, 29–35. 10.1016/j.ejps.2014.06.024 25010261

[B22] Joshi B. Bastiani M. Strugnell S. S. Boscher C. Parton R. G. Nabi I. R. (2012). Phosphocaveolin-1 is a mechanotransducer that induces caveola biogenesis via Egr1 transcriptional regulation. J. Cell Biol. 199, 425–435. 10.1083/jcb.201207089 23091071 PMC3483133

[B23] Kabayama H. Nakamura T. Takeuchi M. Iwasaki H. Taniguchi M. Tokushige N.. (2009). Ca^2+^ induces macropinocytosis via F-actin depolymerization during growth cone collapse. Mol. Cell. Neurosci. 40, 27–38. 10.1016/j.mcn.2008.08.009 18848894

[B24] Kitambi S. S. Toledo E. M. Usoskin D. Wee S. Harisankar A. Svensson R.. (2014). Vulnerability of glioblastoma cells to catastrophic vacuolization and death induced by a small molecule. Cell 157, 313–328. 10.1016/j.cell.2014.02.021 24656405

[B27] Lee H. J. Diaz M. F. Price K. M. Ozuna J. A. Zhang S. Sevick-Muraca E. M.. (2017). Fluid shear stress activates YAP1 to promote cancer cell motility. Nat. Commun. 8:14122. 10.1038/ncomms14122 28098159 PMC5253685

[B26] Le X. T. Pham H. T. Do P. T. Fujiwara H. Tanaka K. Li F.. (2013). *Bacopa monnieri* ameliorates memory deficits in olfactory bulbectomized mice: possible involvement of glutamatergic and cholinergic systems. Neurochem. Res. 38, 2201–2215. 10.1007/s11064-013-1129-6 23949198

[B29] Li L. Hanahan D. (2013). Hijacking the neuronal NMDAR signaling circuit to promote tumor growth and invasion. Cell 153, 86–100. 10.1016/j.cell.2013.02.051 23540692

[B28] Li B. Zhang S. Zhang H. Hertz L. Peng L. (2011). Fluoxetine affects GluK2 editing, glutamate-evoked Ca^2+^ influx and extracellular signal-regulated kinase phosphorylation in mouse astrocytes. J. Psychiatry Neurosci. 36, 322–338. 10.1503/jpn.100094 21320410 PMC3163648

[B30] Lima S. Milstien S. Spiegel S. (2017). Sphingosine and sphingosine kinase 1 involvement in endocytic membrane trafficking. J. Biol. Chem. 292, 3074–3088. 10.1074/jbc.M116.762377 28049734 PMC5336145

[B31] Lisman J. Yasuda R. Raghavachari S. (2012). Mechanisms of CaMKII action in long-term potentiation. Nat. Rev. Neurosci. 13, 169–182. 10.1038/nrn3192 22334212 PMC4050655

[B32] Ma J. Lim C. Sacher J. R. Van Houten B. Qian W. Wipf P. (2015). Mitochondrial targeted β-lapachone induces mitochondrial dysfunction and catastrophic vacuolization in cancer cells. Bioorg. Med. Chem. Lett. 25, 4828–4833. 10.1016/j.bmcl.2015.06.073 26159482 PMC4607627

[B33] Majumdar S. Basu A. Paul P. Halder M. Jha S. (2013). “Bacosides and neuroprotection,” in Natural Products: Phytochemistry, Botany and Metabolism of Alkaloids, Phenolics and Terpenes, eds Ramawat K. G. Mérillon J.-M. (Berlin, Heidelberg: Springer), 3639–3660.

[B34] Mallick M. N. Akhtar M. S. Najm M. Z. Tamboli E. T. Ahmad S. Husain S. A. (2015). Evaluation of anticancer potential of *Bacopa monnieri* L. against MCF-7 and MDA-MB 231 cell line. J. Pharm. Bioallied Sci. 7, 325–328. 10.4103/0975-7406.168038 26681894 PMC4678980

[B35] Maltese W. A. Overmeyer J. H. (2014). Methuosis: nonapoptotic cell death associated with vacuolization of macropinosome and endosome compartments. Am. J. Pathol. 184, 1630–1642. 10.1016/j.ajpath.2014.02.028 24726643 PMC4044715

[B36] Marquard J. Otter S. Welters A. Stirban A. Fischer A. Eglinger J.. (2015). Characterization of pancreatic NMDA receptors as possible drug targets for diabetes treatment. Nat. Med. 21, 363–372. 10.1038/nm.3822 25774850

[B37] Maschio M. (2012). Brain tumor-related epilepsy. Curr. Neuropharmacol. 10, 124–133. 10.2174/157015912800604470 23204982 PMC3386502

[B38] McCoy F. Darbandi R. Lee H. C. Bharatham K. Moldoveanu T. Grace C. R.. (2013). Metabolic activation of CaMKII by coenzyme A. Mol. Cell 52, 325–339. 10.1016/j.molcel.2013.08.043 24095281 PMC3967247

[B39] Messam C. A. Hou J. Gronostajski R. M. Major E. O. (2003). Lineage pathway of human brain progenitor cells identified by JC virus susceptibility. Ann. Neurol. 53, 636–646. 10.1002/ana.10523 12730998

[B40] Mironova E. V. Evstratova A. A. Antonov S. M. (2007). A fluorescence vital assay for the recognition and quantification of excitotoxic cell death by necrosis and apoptosis using confocal microscopy on neurons in culture. J. Neurosci. Methods 163, 1–8. 10.1016/j.jneumeth.2007.02.010 17395268

[B41] Morgan A. Stevens J. (2010). Does *Bacopa monnieri* improve memory performance in older persons? Results of a randomized, placebo-controlled, double-blind trial. J. Altern. Complement. Med. 16, 753–759. 10.1089/acm.2009.0342 20590480

[B25] Nandagaon V. S. Kulkarni A. R. (2013). *In vitro* antioxidant and cytotoxicity activitiy of *Bacopa monnieri* and baliospermum montanum muell arg. Int. J. Pharm. Appl. 4, 63–67.

[B42] Nathan P. J. Clarke J. Lloyd J. Hutchison C. W. Downey L. Stough C. (2001). The acute effects of an extract of *Bacopa monniera* (Brahmi) on cognitive function in healthy normal subjects. Hum. Psychopharmacol. 16, 345–351. 10.1002/hup.306 12404571

[B43] Overmeyer J. H. Kaul A. Johnson E. E. Maltese W. A. (2008). Active ras triggers death in glioblastoma cells through hyperstimulation of macropinocytosis. Mol. Cancer Res. 6, 965–977. 10.1158/1541-7786.MCR-07-2036 18567800 PMC2994605

[B44] Overmeyer J. H. Young A. M. Bhanot H. Maltese W. A. (2011). A chalcone-related small molecule that induces methuosis, a novel form of non-apoptotic cell death, in glioblastoma cells. Mol. Cancer 10:69. 10.1186/1476-4598-10-69 21639944 PMC3118192

[B45] Ozawa T. (2010). Modulation of ryanodine receptor Ca^2+^ channels (Review). Mol. Med. Rep. 3, 199–204. 10.3892/mmr_00000240 21472222

[B1] Patil A. Vadera K. Patil D. Phatak A. Juvekar A. Chandra N. (2014). *In vitro* anticancer activity and phytochemical analysis of *Bacopa monnieri* (L.) wettst. Int. J. Pharm. Sci. Res. 5, 4432–4438. 10.13040/IJPSR.0975-8232.5(10).4432-38

[B46] Parton R. G. del Pozo M. A. (2013). Caveolae as plasma membrane sensors, protectors and organizers. Nat. Rev. Mol. Cell Biol. 14, 98–112. 10.1038/nrm3512 23340574

[B47] Pasparakis M. Vandenabeele P. (2015). Necroptosis and its role in inflammation. Nature 517, 311–320. 10.1038/nature14191 25592536

[B48] Pellicena P. Schulman H. (2014). CaMKII inhibitors: from research tools to therapeutic agents. Front. Pharmacol. 5:21. 10.3389/fphar.2014.00021 24600394 PMC3929941

[B49] Pisha E. Chai H. Lee I.-S. Chagwedera T. E. Farnsworth N. R. Cordell G. A.. (1995). Discovery of betulinic acid as a selective inhibitor of human melanoma that functions by induction of apoptosis. Nat. Med. 1, 1046–1051. 10.1038/nm1095-1046 7489361

[B50] Prakash N. S. Sundaram R. Mitra S. K. (2011). *In vitro* and *In vivo* anticancer activity of bacoside a from whole plant of *Bacopa monnieiri* (Linn). Am. J. Pharmacol. Toxicol. 6, 11–19. 10.3844/ajptsp.2011.11.19

[B51] Prisila Dulcy C. Singh H. K. Preethi J. Rajan K. E. (2012). Standardized extract of Bacopa monniera (BESEB CDRI-08) attenuates contextual associative learning deficits in the aging rat’s brain induced by D-galactose. J. Neurosci. Res. 90, 2053–2064. 10.1002/jnr.23080 22715050

[B52] Reyes-Reyes E. M. Salipur F. R. Shams M. Forsthoefel M. K. Bates P. J. (2015). Mechanistic studies of anticancer aptamer AS1411 reveal a novel role for nucleolin in regulating Rac1 activation. Mol. Oncol. 9, 1392–1405. 10.1016/j.molonc.2015.03.012 25911416 PMC4523413

[B53] Sadowski Ł. Jastrzbski K. Kalaidzidis Y. Heldin C. H. Hellberg C. Miaczynska M. (2013). Dynamin inhibitors impair endocytosis and mitogenic signaling of PDGF. Traffic 14, 725–736. 10.1111/tra.12061 23425318 PMC3712465

[B54] Sinha B. Köster D. Ruez R. Gonnord P. Bastiani M. Abankwa D.. (2011). Cells respond to mechanical stress by rapid disassembly of caveolae. Cell 144, 402–413. 10.1016/j.cell.2010.12.031 21295700 PMC3042189

[B55] Suresh S. (2007). Biomechanics and biophysics of cancer cells. Acta Biomater. 3, 413–438. 10.1016/j.actamat.2007.04.022 17540628 PMC2917191

[B56] Taddei M. L. Giannoni E. Fiaschi T. Chiarugi P. (2012). Anoikis: an emerging hallmark in health and diseases. J. Pathol. 226, 380–393. 10.1002/path.3000 21953325

[B57] Wang Y. Y. Zhao R. Zhe H. (2015). The emerging role of CaMKII in cancer. Oncotarget 6, 11725–11734. 10.18632/oncotarget.3955 25961153 PMC4494900

[B58] Yadav K. D. Reddy K. R. C. (2013). Critical review on pharmacological properties of Brahmi. Int. J. Ayurvedic Med. 4, 92–99.

[B59] Yeh Y. C. Tang M. J. Parekh A. B. (2014). Caveolin-1 alters the pattern of cytoplasmic Ca^2+^ oscillations and Ca^2+^-dependent gene expression by enhancing leukotriene receptor desensitization. J. Biol. Chem. 289, 17843–17853. 10.1074/jbc.M114.553453 24755228 PMC4067216

[B60] Yoshida N. Takada T. Yamamura Y. Adachi I. Suzuki H. Kawakami J. (2008). Inhibitory effects of terpenoids on multidrug resistance-associated protein 2- and breast cancer resistance protein-mediated transport. Drug Metab. Dispos. 36, 1206–1211. 10.1124/dmd.107.019513 18436619

[B61] Zimnicka A. M. Husain Y. S. Shajahan A. N. Sverdlov M. Chaga O. Chen Z.. (2016). Src-dependent phosphorylation of caveolin-1 Tyr-14 promotes swelling and release of caveolae. Mol. Biol. Cell 27, 2090–2106. 10.1091/mbc.e15-11-0756 27170175 PMC4927282

