# Supplementary material for: Bacoside A Induces Tumor Cell Death in Human Glioblastoma Cell Lines through Catastrophic Macropinocytosis
Source: Front Mol Neurosci. 2017 Jun 15;10:171. doi: 10.3389/fnmol.2017.00171 (PMC5471305; doi:10.3389/fnmol.2017.00171)
Supplement: Supplementary file 1 [file Data_Sheet_1.pdf]

SUPPLEMENTARY FIGURES
S1-S9 WITH LEGENDS

Research Article Title:

Bacoside A Induces Tumor Cell Death In Human Glioblastoma Cell Lines Through Catastrophic Macropinocytosis

Authors:

Sebastian John[1], K.C. Sivakumar[2] and Rashmi Mishra[1]*

Authors Affiliations:

[1]*Disease Biology Program, Department of Neurobiology and Genetics, Rajiv Gandhi Centre for Biotechnology, Thycaud PO, Poojappura, Thiruvananthapuram, India-695014.*

[2]*Distributed Information Sub-Centre, Rajiv Gandhi Centre for Biotechnology, Thycaud PO, Poojappura, Thiruvananthapuram, India-695014.*

*Corresponding author: Rashmi Mishra, Email: rashmimishra@rgcb.res.in, Tel- +91- 8750347930

SUPPLEMENTARY FIGURE LEGENDS

S1 Fig: CaMK2 isoforms gene expression in different types of gliomas.

A), B), C) and **D)** shows REMBRANT data based comparative gene expression profiles of CaMK2A, 2B, 2D and 2G isoforms of CaMK2 enzyme in normal and glioma patients sub-classified into glioblastoma multiforme (GBM), oligodendroglioma and astrocytoma. Data revealed that all isoforms were expressed at a lower level than in normal controls. CaMK2D levels were not much affected in gliomas versus normal subjects. CaMK2G gene expression was highest in all subtypes in comparison to other isoforms. REMBRANT data was extracted from URL: www.betastasis.com

S2 Fig: CaMK2 isoforms gene expression associated survival curves in Mesenchymal and Neural glioblastoma multiforme (GBM) patients.

A-F) Median survival curve analysis using glioblastoma patient data in TCGA data set under mesenchymal and neural (occurs mainly in old age, infrequent) GBM subtypes revealed that CaMK2G expression alone were significantly associated with prognosis in neural subtype [median survival, 18.5 months in CaMK2G high group (red) versus survival in CaMK2G low group (blue)]. TCGA data was extracted from URL: www.betastasis.com, keeping median range for total GBM patient cohort.

S3 Fig: CaMK2 isoforms gene expression associated survival curves in Pro-neural glioblastoma multiforme (GBM) patients.

A-C) Median survival curve analysis using glioblastoma patient data in TCGA data set under proneural (occurrence infrequent) GBM subtypes revealed no significant association of CaMK2 isoforms with prognosis in CaMK2 high group (red) versus CaMK2 low group (blue). TCGA data was extracted from URL: www.betastasis.com, keeping median range for total GBM patient cohort.

S4 Fig: CaMK2A phosphorylation at T286 but not non-phosphorylated CaMK2A is reduced in more advanced stages of pediatric GBM tumors.

GBM patient tissue array immunostaining data showed significantly higher levels of non-phospho CaMK2A protein levels to be associated with pediatric GBM grade IV but reverse trend was noticed for proportion of CaMK2A phosphorylated at T286.

S5 Fig: *Bacopa monnieri* (BM) administration induces extreme cytoplasmic vacuolization in LN229 glioblastoma cells.

A) LN229 glioblastoma tumor cell, upon BM treatment (100µg/ml), showed cell surface ruffling (magenta arrowheads) and formation of cup shaped structure (red arrows) which are characteristic of the initial steps in the biogenesis of macropinosomes. By about 7-12 hours post treatment, LN229 glioblastoma cells showed phase lucent vacuoles/macropinosomes that progressively fused to generate larger vesicles. **B)** Live cell dextran-TMR uptake assays for detection/identification of macropinocytotic vesicles showed significantly more numerous and larger dextran-TMR filled macropinosomes (white arrows) in BM treated versus untreated cells.

S6 Fig: Snapshot of the *in silico* docking and binding analysis of various components of *Bacopa monnieri* extract to CaMK2A enzyme

A-F) shows docked model of individual components of the extract of *Bacopa monnieri* to CaMK2A enzyme.

S7 Fig: Snapshot of the *in silico* interaction of residues in Bacoside A-CaMK2A docked model.

Magenta arrows highlight the hydrogen bond interactions between Bacoside A sugar moieties with CaMK2A protein residues. Arabinose is observed to interact with Thr310 and Glu216, whereas glucose interacts with Thr310.

S8 Fig: Bacoside A treatment has similar effects on cell morphology in micro-environmental acidification induced temozolomide resistant and parental LN229 GBM cells.

A) MTT assay to estimate the optical density due to LN229 tumor cell growth at different extracellular pH (pH 7.4, 6.2 and 3.4) in presence or absence of anti-GBM FDA approved drug, temozolomide (TMZ), showed a loss of anti-survival activity of TMZ at lower pHs (chemoresistance). **B-F)** Bacoside A induced macropinocytotic phenotype leading to cell death and its vacuolization induced cytotoxic activity was unaffected in extracellular pH gradients, suggesting no loss in activity at the determined effective dose of 8μg/ml. Error bar=S.D. The analysis is representative of 3 independent experiments.

S9 Fig: Autophagosome marker LC3II levels are unchanged with Bacoside A treatment.

A-C) No significant increase in LC3II, an autophagosome marker was observed in Bacoside A treated versus untreated cells. The membranes of enlarged macropinocytotic vesicles formed upon Bacoside A treatment did not show localization of autophagosome marker LC3II (red arrows and inset, LC3II positivity can be seen only around but not on the vesicle membrane)

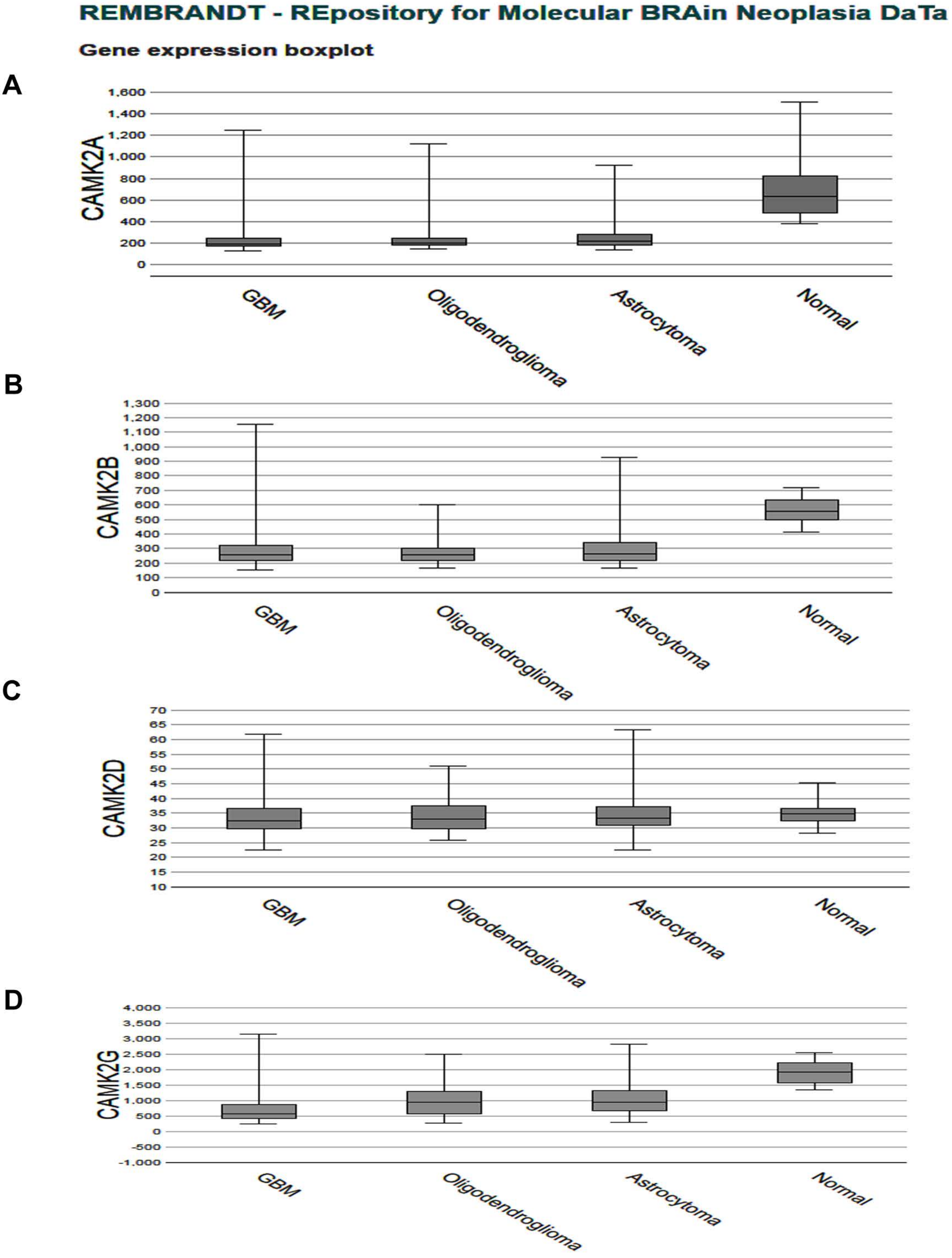
REMBRANDT - REpository for Molecular BRAin Neoplasia DaTa
Gene expression boxplot
A
CAMK2A
1,600
1,400
1,200
1,000
800
600
400
200
0
GBM
Oligodendroglioma
Astrocytoma
Normal
B
CAMK2B
1,300
1,200
1,100
1,000
900
800
700
600
500
400
300
200
100
0
GBM
Oligodendroglioma
Astrocytoma
Normal
C
CAMK2D
70
65
60
55
50
45
40
35
30
25
20
15
10
GBM
Oligodendroglioma
Astrocytoma
Normal
D
CAMK2G
4,000
3,500
3,000
2,500
2,000
1,500
1,000
500
0
-500
-1,000
GBM
Oligodendroglioma
Astrocytoma
Normal

S1 Fig

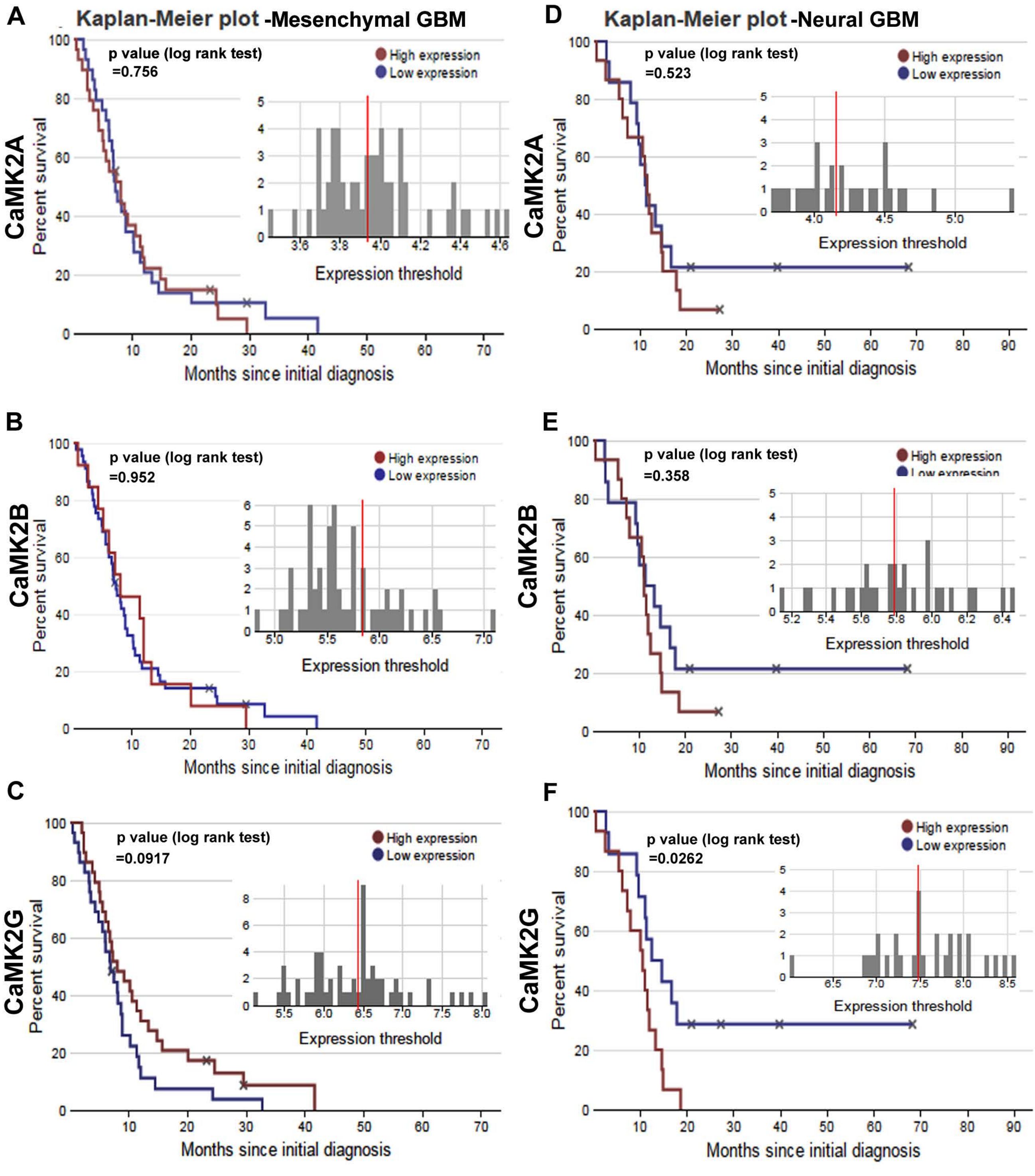
A
Kaplan-Meier plot -Mesenchymal GBM
CaMK2A
p value (log rank test) =0.756
High expression
Low expression
Percent survival
Expression threshold
Months since initial diagnosis
B
CaMK2B
p value (log rank test) =0.952
High expression
Low expression
Percent survival
Expression threshold
Months since initial diagnosis
C
CaMK2G
p value (log rank test) =0.0917
High expression
Low expression
Percent survival
Expression threshold
Months since initial diagnosis
D
Kaplan-Meier plot -Neural GBM
CaMK2A
p value (log rank test) =0.523
High expression
Low expression
Percent survival
Expression threshold
Months since initial diagnosis
E
CaMK2B
p value (log rank test) =0.358
High expression
Percent survival
Expression threshold
Months since initial diagnosis
F
CaMK2G
p value (log rank test) =0.0262
High expression
Low expression
Percent survival
Expression threshold
Months since initial diagnosis

S2 Fig

Kaplan-Meier plot-ProNeural GBM

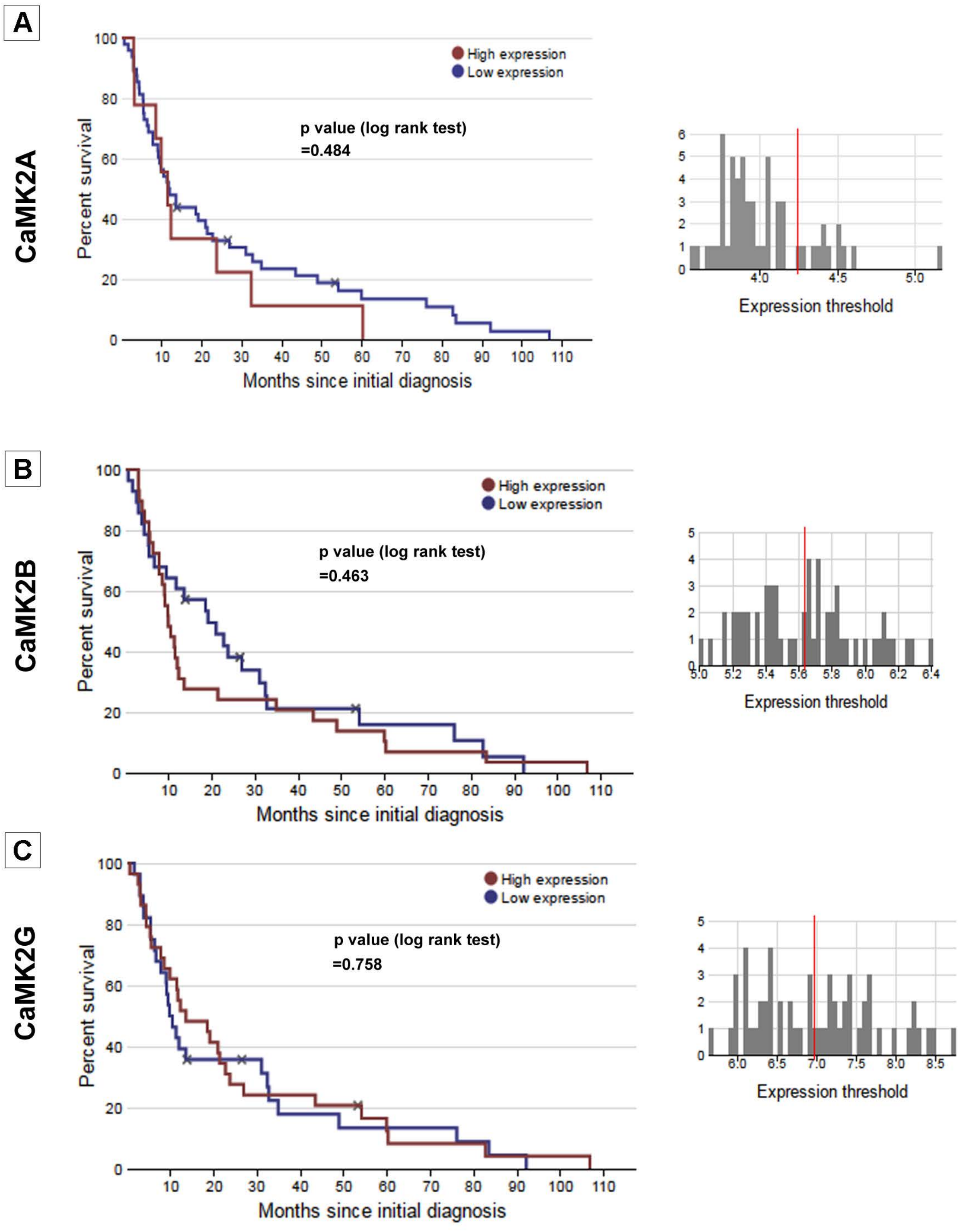

S3 Fig

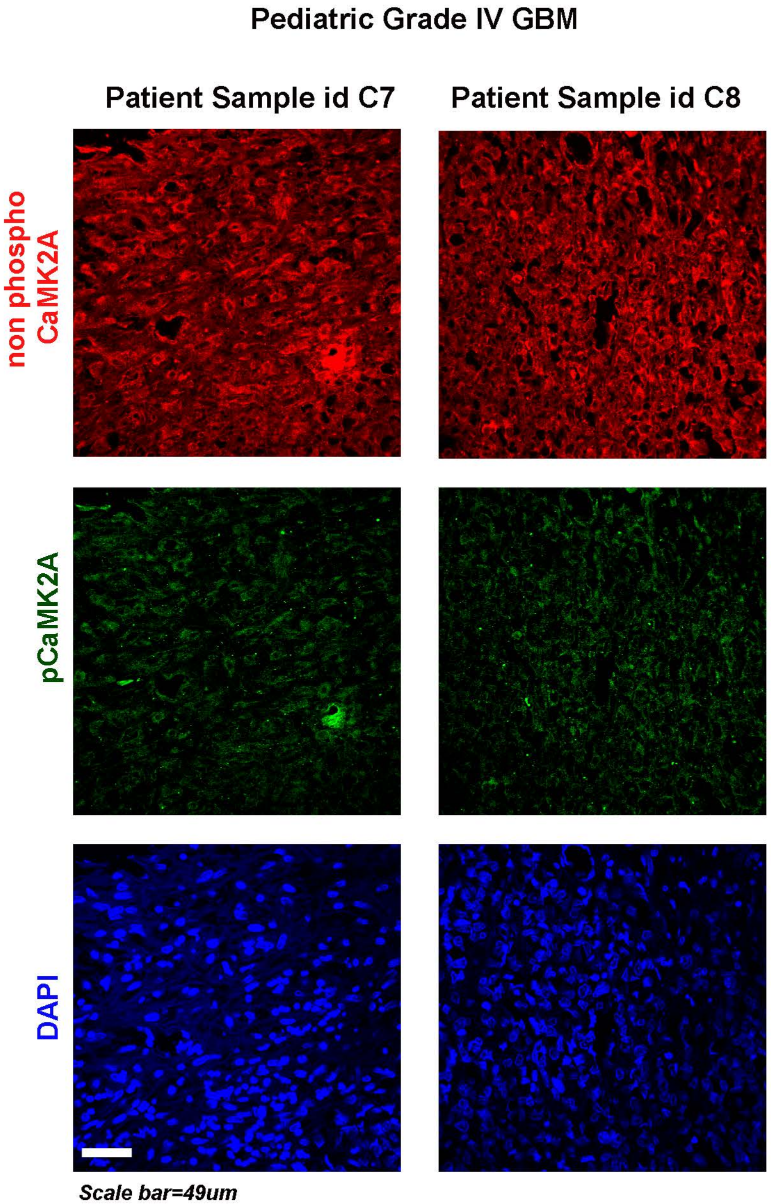
Pediatric Grade IV GBM
Patient Sample id C7
Patient Sample id C8
non phospho CaMK2A
pCaMK2A
DAPI
Scale bar=49um

S4 Fig

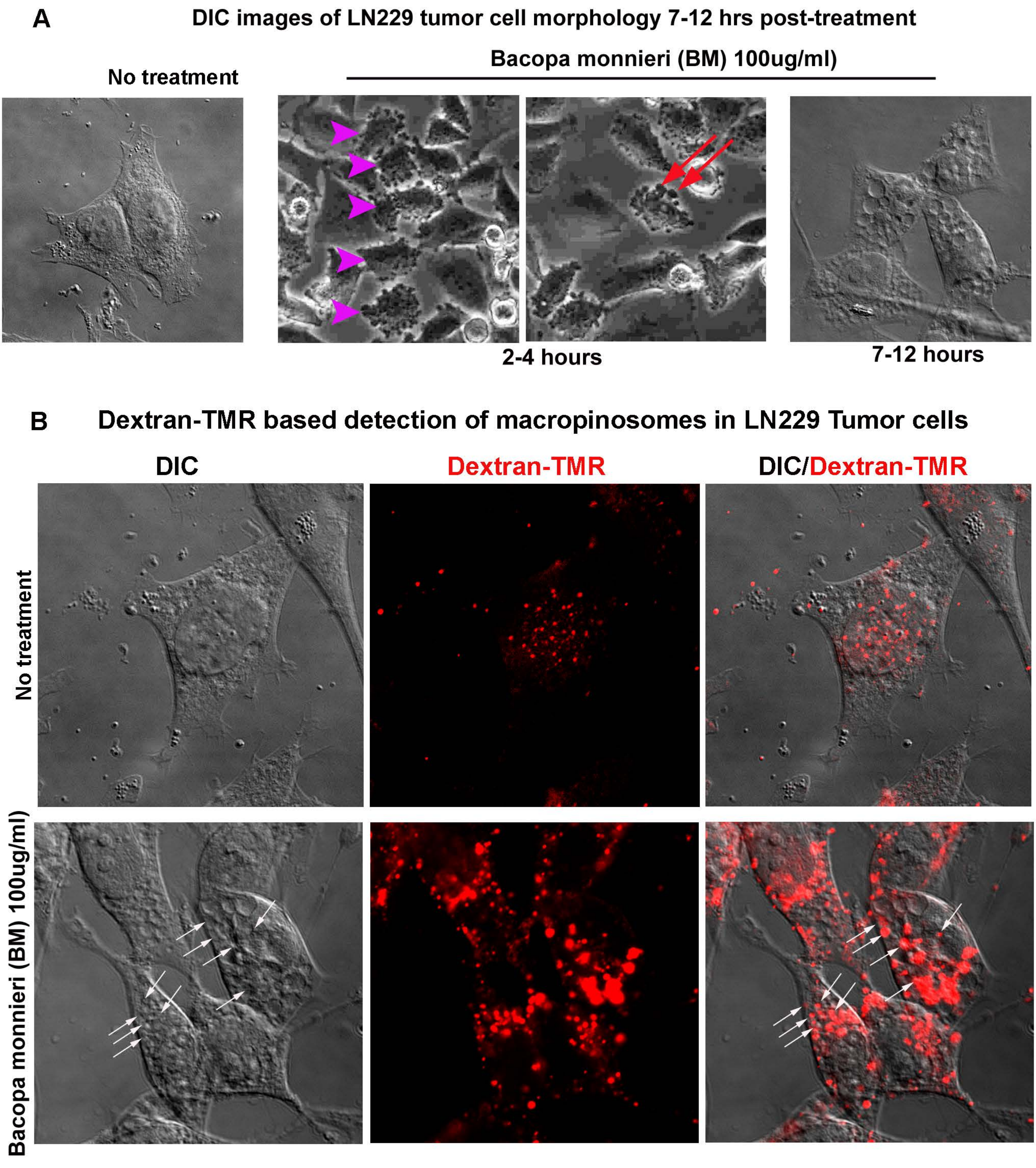
A
DIC images of LN229 tumor cell morphology 7-12 hrs post-treatment
No treatment
Bacopa monnieri (BM) 100ug/ml)
2-4 hours
7-12 hours
B
Dextran-TMR based detection of macropinosomes in LN229 Tumor cells
DIC
Dextran-TMR
DIC/Dextran-TMR
No treatment
Bacopa monnieri (BM) 100ug/ml)

S5 Fig

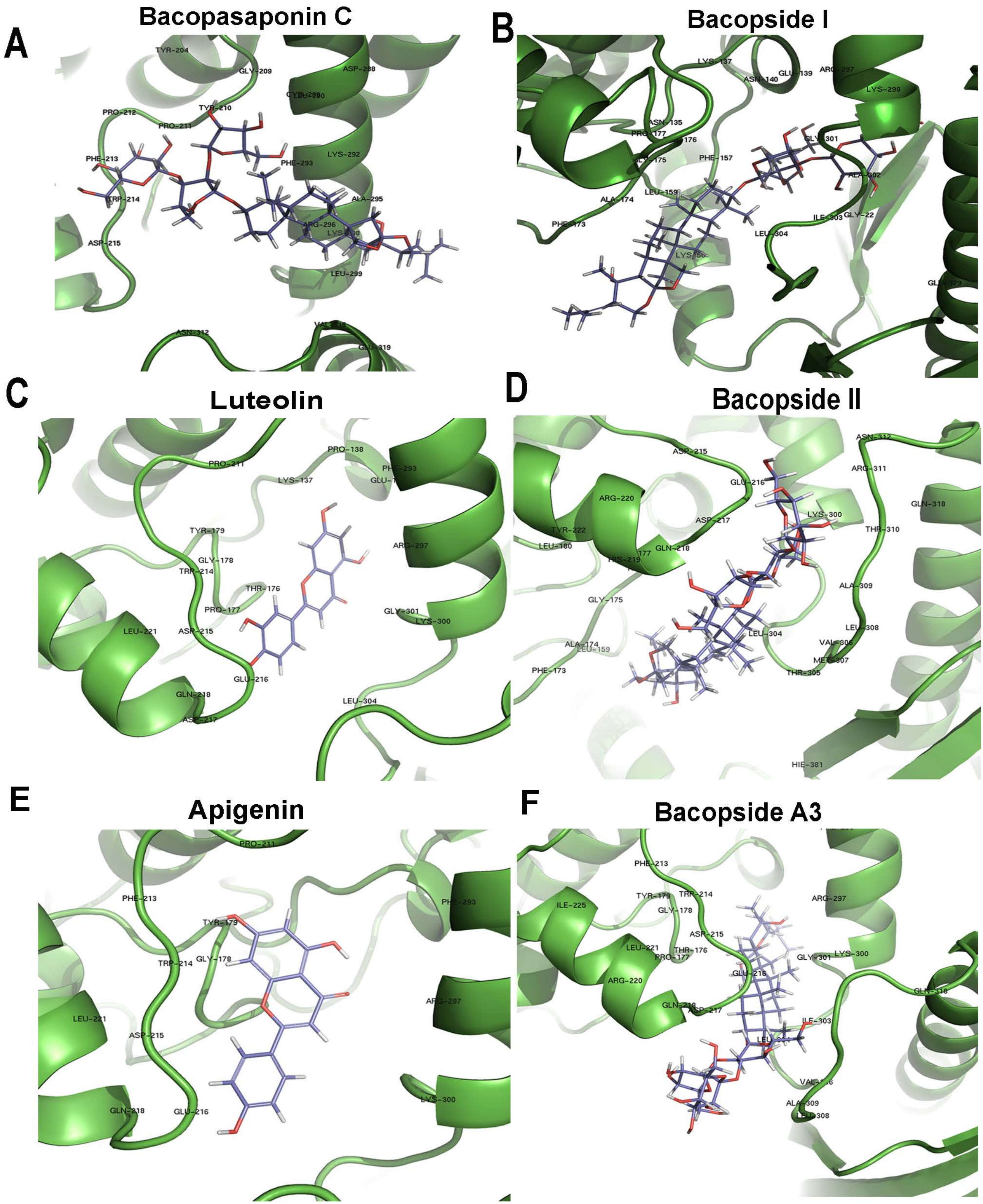
A
Bacopasaponin C
TYR-204
GLY-209
ASP-288
TYR-210
PRO-212
PRO-211
PHE-213
LYS-292
PHE-293
TRP-214
ALA-295
ARG-296
ASP-215
LEU-299
ASN-312
GLU-319
B
Bacopside I
LYS-137
ASN-140
GLU-139
LYS-298
ASN-135
PRO-177
GLY-301
PHE-157
GLY-175
ALA-302
LEU-159
ALA-174
PHE-173
ILE-303
LEU-304
C
Luteolin
PRO-138
PRO-211
LYS-137
TYR-179
GLY-178
ARG-297
TRP-214
THR-176
PRO-177
GLY-301
LYS-300
LEU-221
ASP-215
GLU-216
GLN-218
LEU-304
D
Bacopside II
ASN-312
ASP-215
ARG-311
GLU-216
ARG-220
GLN-318
LYS-300
ASP-217
THR-310
TYR-222
LEU-180
GLN-218
HIS-219
GLY-175
ALA-309
LEU-308
LEU-304
ALA-174
LEU-159
PHE-173
THR-305
HIE-381
E
Apigenin
PRO-211
PHE-213
PHE-293
TYR-179
TRP-214
GLY-178
ARG-297
LEU-221
ASP-215
GLN-218
GLU-216
LYS-300
F
Bacopside A3
PHE-213
TYR-179
TRP-214
ARG-297
ILE-225
GLY-178
ASP-215
LEU-221
THR-176
GLY-301
LYS-300
PRO-177
ARG-220
GLU-216
GLN-318
ASP-217
ILE-303
ALA-309
LEU-308

S6 Fig

Bacoside A-CaMK2A docked model

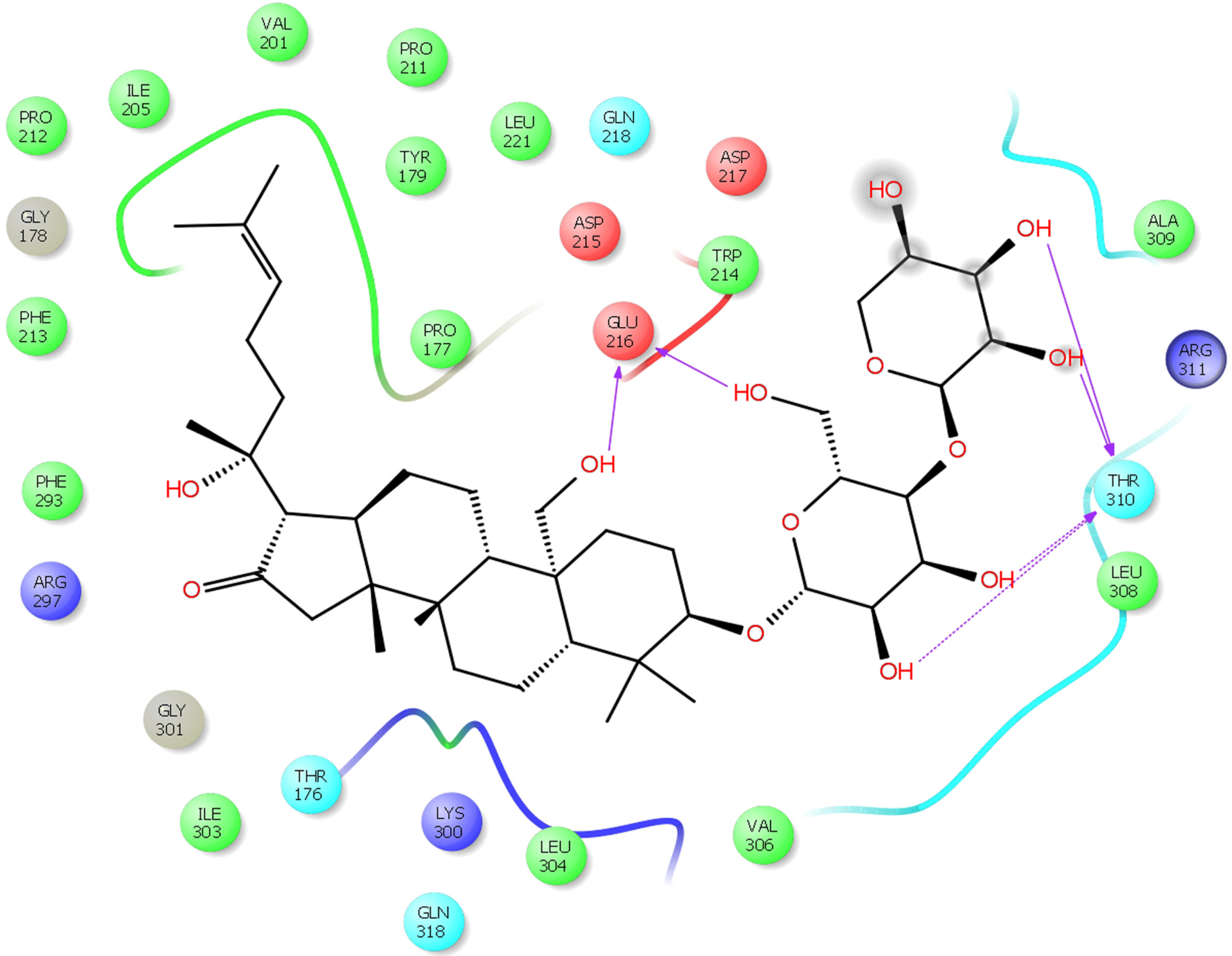

S7 Fig

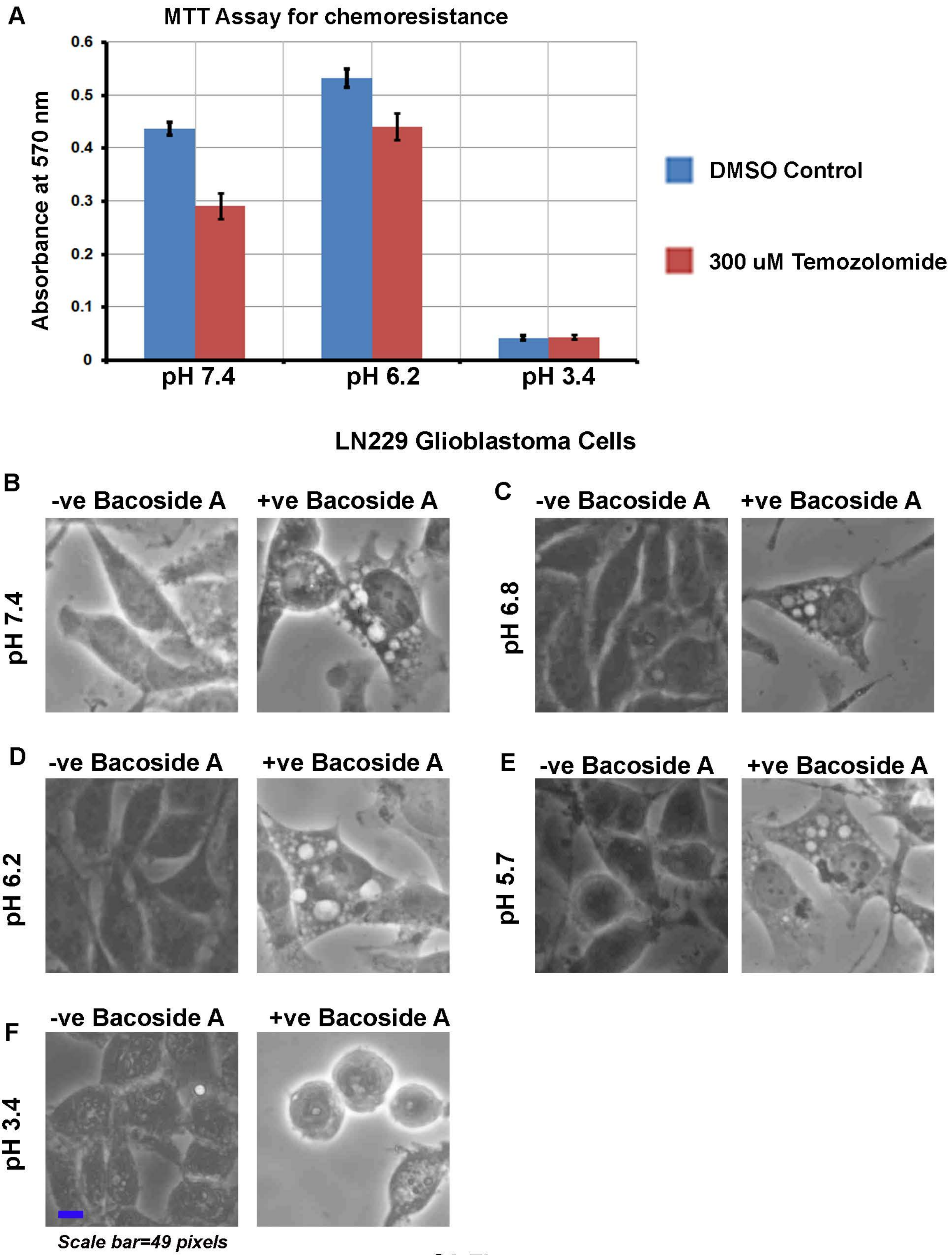
A
MTT Assay for chemoresistance
Absorbance at 570 nm
0.6
0.5
0.4
0.3
0.2
0.1
0
pH 7.4
pH 6.2
pH 3.4
DMSO Control
300 uM Temozolomide
LN229 Glioblastoma Cells
B
-ve Bacoside A
+ve Bacoside A
pH 7.4
C
-ve Bacoside A
+ve Bacoside A
pH 6.8
D
-ve Bacoside A
+ve Bacoside A
pH 6.2
E
-ve Bacoside A
+ve Bacoside A
pH 5.7
F
-ve Bacoside A
+ve Bacoside A
pH 3.4
Scale bar=49 pixels

S8 Fig

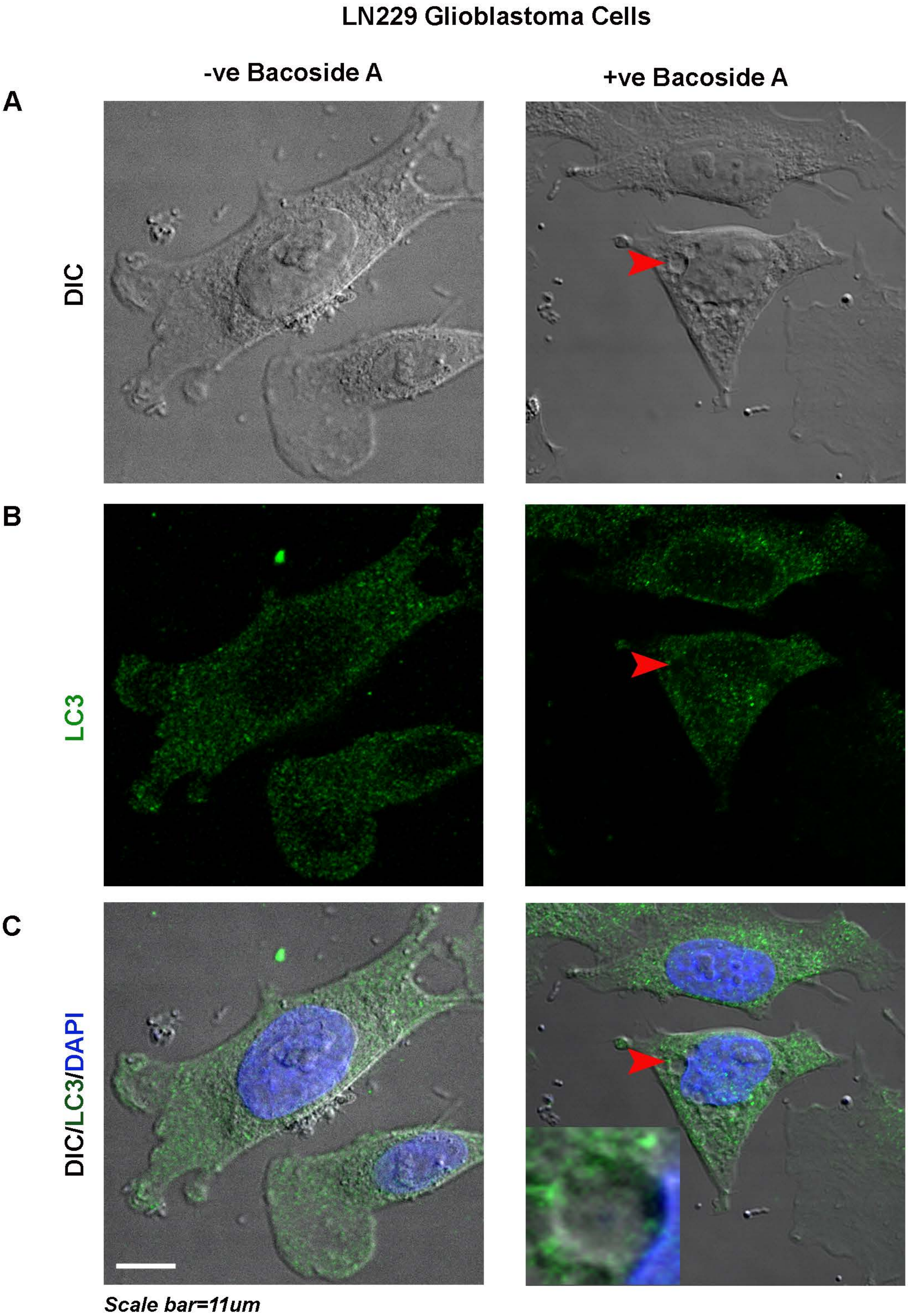
LN229 Glioblastoma Cells
-ve Bacoside A
+ve Bacoside A
A
B
C
DIC
LC3
DIC/LC3/DAPI
Scale bar=11um

S9 Fig
